# Targeting the Rift Valley Fever Virus Polymerase: Resistance Mechanisms and Structural Insights

**DOI:** 10.1021/acsinfecdis.5c00832

**Published:** 2025-10-30

**Authors:** Michal Král’, Amiyaranjan Das, Tomáš Kotačka, Anna Blahošová, Veronika Liščáková, Jan Hodek, Jan Konvalinka, Gabriel Demo, Milan Kožíšek

**Affiliations:** † 89220 Institute of Organic Chemistry and Biochemistry of the Czech Academy of Sciences, Flemingovo n. 2, 166 10 Prague 6, Czech Republic; ‡ First Faculty of Medicine, Charles University, Kateřinská 1660, 121 08 Prague 2, Czech Republic; § Central European Institute of Technology, Masaryk University, Kamenice 753/5, 625 00 Brno, Czech Republic; ∥ National Centre for Biomolecular Research, Faculty of Science, Masaryk University, Kamenice 5, 625 00 Brno, Czech Republic; ⊥ Department of Biology, Faculty of Science, Charles University, Viničná 7, 128 00 Prague 2, Czech Republic; # Department of Biochemistry, Faculty of Science, Charles University, Hlavova 8, 128 00 Prague 2, Czech Republic

**Keywords:** Rift Valley fever virus, L protein, antivirals, polymerase inhibitors, resistance mutations, structural insights

## Abstract

Rift Valley fever virus (RVFV) is an arbovirus from the *Phenuiviridae* family that can cause severe disease in humans and livestock, with outbreaks resulting in substantial economic losses. Despite the availability of attenuated vaccines for animals, there is no approved preventive or therapeutic agent for human RVFV infections. Moreover, the safety and efficacy of the current veterinary vaccines remain uncertain. The RVFV L protein, a 250 kDa polymerase, plays a key role in viral replication and transcription, containing endonuclease, RNA-dependent RNA polymerase (RdRp), and cap-binding domains. Structurally conserved across related viruses and functionally analogous to the influenza virus polymerase, the L protein is a compelling antiviral target. In our study, we screened a library of polymerase inhibitors and identified several compounds with inhibitory activity against the RVFV polymerase. We validated their effect using both live virus assays and a minigenome luciferase reporter system. Resistance mutants were generated, and key mutations conferring resistance to the inhibitors were identified and characterized. Some of these key mutations were structurally analyzed via cryo-electron microscopy, using a new structure of the apo form of wild-type RVFV L protein resolved at 3.5 Å. This structure provides critical insights into how the mutations can influence inhibitor binding and RVFV polymerase function. These findings provide insight into how these mutations may confer resistance by affecting inhibitor binding and polymerase activity.

## Graphical abstract

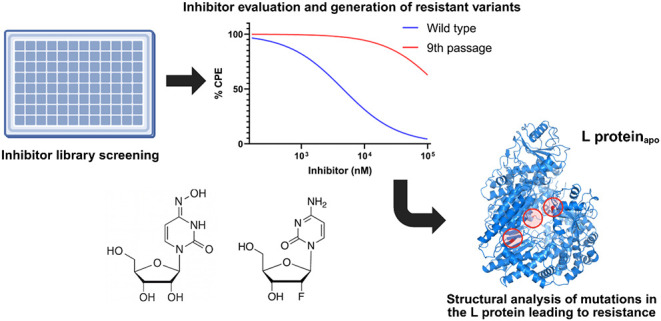

Rift Valley fever virus (RVFV) is a mosquito-borne phlebovirus of the *Hareavirales* order (formerly *Bunyavirales*) that causes severe disease in humans and livestock. Outbreaks result in high livestock mortality, nearly 100% abortion rates in pregnant animals, and major economic losses.^1^ In humans, infection usually presents as a mild, febrile illness, but approximately 2% of cases progress to severe disease with high mortality or long-term complications, including encephalitis, hepatitis, hemorrhagic fever, or ocular damage leading to blindness.^2^ Limited data also indicate an increased risk of stillbirth during pregnancy.^3^ The economic impact of outbreaks is substantial, with losses estimated at $330 million in Somalia (1998–2003), $471 million in Somalia (2006–2007), $66 million in Kenya, $10 million in Saudi Arabia, and $107 million in Yemen.^4^ Currently, no approved treatment exists; veterinary vaccines are available but restricted due to safety and efficacy concerns.^5^ Consequently, RVFV is recognized by the World Health Organization as a priority pathogen.^6^

The replication and transcription of RVFV, like other viruses in the *Hareavirales* order, are mediated by the L protein, a 250 kDa RNA-dependent RNA polymerase (RdRp). The L protein contains three domains: endonuclease (ENDO), RdRp, and C-terminal domain (including the cap-binding domain), organized in a similar manner to the PA-PB1-PB2 heterotrimeric complex of influenza virus.^7^ The structural conservation of the L protein within the virus family makes it a promising antiviral target.^8^ Viral polymerases are essential for replication and transcription across diverse genome types, and their highly conserved catalytic cores—comprising “finger,” “palm,” and “thumb” subdomains, responsible for template recognition, nucleotide discrimination, and catalysis—highlight their suitability for broad-spectrum antiviral development.^9,10^

Several FDA-approved drugs target both DNA- and RNA-dependent viral polymerases, which are broadly classified as nucleoside, nucleotide, or non-nucleoside inhibitors. These can be further divided by their mechanism of action. Obligate chain terminators lack the 3′-hydroxyl group needed for the next phosphodiester bond, thereby halting elongation once incorporated.^11^ Non-obligate chain terminators allow limited extension after incorporation but markedly reduce elongation efficiency.^11^ Another strategy is the use of mutagenic nucleotides, which exploit the absence of RNA repair mechanisms by introducing ambiguous base-pairing, leading to lethal mutagenesis; ribavirin, favipiravir, and molnupiravir are FDA-approved examples, particularly effective against exonuclease-proficient viruses such as SARS-CoV-2.^12^

As no FDA- or EMA-approved inhibitors currently target bunyaviruses, treatment remains limited to supportive care. A few antivirals have shown promise *in vitro* or in animal models. Ribavirin has been tested against several bunyaviruses, including Crimean-Congo hemorrhagic fever virus (CCHFV), arenaviruses, and hantaviruses, but its efficacy and safety are inconsistent, with limited benefit except in early phase CCHFV infection and some Andes hantavirus models.^13–16^ Favipiravir (T-705), a more potent RdRp inhibitor, displays broad activity against arenaviruses, CCHFV, and RVFV, with demonstrated efficacy in both cell culture and animal models.^17–20^ It is also highly effective against severe fever with thrombocytopenia syndrome virus (SFTSV) and is undergoing clinical trials for this indication.^21^ Other candidates include 2′-fluoro-2′-deoxycytidine (2′-FdC), which inhibits CCHFV, Lassa virus (LASV), and multiple phleboviruses including RVFV, SFTSV, and Heartland virus,^22–24^ and galidesivir, an adenosine analog that protects hamsters from lethal RVFV infection.^25^ Development of bunyaviral RdRp inhibitors has been historically hindered by the relative lack of structural data for their L proteins. In recent years, major progress has been made with high-resolution cryo-EM structures of full-length bunyaviral L proteins, including complexes with viral RNA (vRNA), opening new avenues for structure-based inhibitor design.^26–36^

In this work, we screened a panel of polymerase and reverse transcriptase inhibitors as potential inhibitors of the RVFV L protein. Following the screening, resistant variants of the virus emerged that carried mutations associated with a reduced sensitivity to the selected inhibitors. Sequencing these resistant variants allowed us to determine the mutations responsible for their resistance. The cryo-EM structure of wild-type RVFV L protein in its apo form (L protein_apo_) provided a structural framework to analyze the positions of these mutations and their potential effects on the L protein’s function and inhibitor resistance. This analysis highlights how these mutations may impact inhibitor binding and polymerase activity.

## Results and Discussion

### Screening Identifies Potential Inhibitors of RVFV L Protein

To explore various antivirals’ ability to inhibit RVFV infection in tissue cultures, we employed a cytopathic effect reduction assay evaluated using the XTT colorimetric assay. The selected compounds and their inhibitory potencies are summarized in Figure 1.

**Figure 1:**
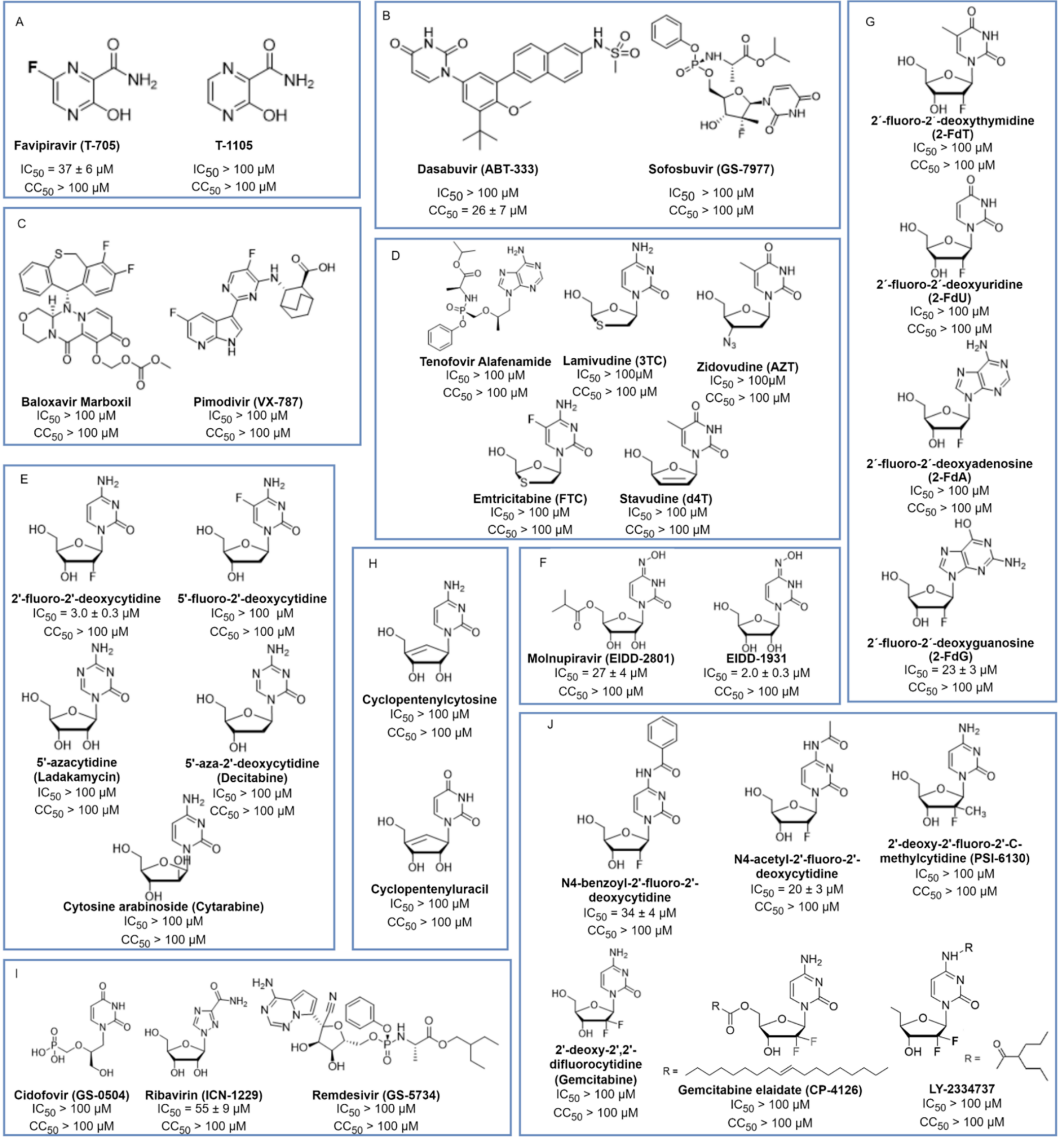
Panel of inhibitors screened for RVFV inhibition. IC_50_ values were determined using the cytopathic effect reduction assay in triplicate, shown in Figures 2 and S1B. The classes of inhibitors were (**A**) Favipiravir and its derivative, (**B**) HCV RdRp inhibitors, (**C**) influenza virus RdRp inhibitors, (**D**) HIV reverse transcriptase inhibitors, (**E**) cytidine and 2′-deoxycytidine derivatives, (**F**) molnupiravir and its active form, (**G**) 2′-fluoro-2′-deoxynucleotides, (**H**) cyclopentenyl-cytosine derivates, (**I**) other nucleotide-based antivirals, and (**J**) 2′-fluoro-2′-deoxycytidine derivatives. CC_50_ values represent the cytotoxic concentrations that reduced the target cell viability by 50% (see Figures 2 and S3).

Although RVFV L protein contains a functionally analogous endonuclease and cap-binding domain, the influenza virus inhibitors baloxavir marboxil, and pimodivir had no effect (IC_50_ > 100 μM) on RVFV in cell culture. The hepatitis C virus (HCV) inhibitors sofosbuvir and dasabuvir lacked activity against the virus, similar to inhibitors targeting HIV reverse transcriptase.^37^ Other clinically relevant nucleotide analogs, including remdesivir and cidofovir, also had no effect (Figure S1A).

However, we identified several nucleotide analogs that inhibited RVFV infection at micromolar concentrations. Ribavirin displayed a weak effect (IC_50_ = 55 ± 9 μM) similar to that of favipiravir, which performed only slightly better (IC_50_ = 37 ± 6 μM) (Figure S1B). Screening of various 2′-fluoro-2′-deoxynucleotides revealed that 2′-fluoro-2′-deoxyguanosine and 2′-FdC blocked infection at low-micromolar concentrations, with IC_50_ = 23 ± 3 and 3.0 ± 0.3 μM, respectively. Exploration of cytidine, 2′-deoxycytidine, and 2′-fluoro-2′-deoxycytidine derivatives did not identify potent inhibitors, with only N4-benzoyl-2′-fluoro-2′-deoxycytidine (IC_50_ = 34 ± 4 μM) and N4-acetyl-2′-fluoro-2′-deoxycytidine (IC_50_ = 20 ± 3 μM) showing antiviral activity. Molnupiravir (EIDD-2801) was able to inhibit the virus in a cell culture (IC_50_ = 27 ± 4 μM), but its potency increased substantially when its active form (EIDD-1931) was used to treat cells prior to infection, with IC_50_ = 2.0 ± 0.3 μM (Figure S1B).

To further assess the activity of the best hits (2′-FdC and EIDD-1931) on RVFV replication, we employed a minigenome system based on a luciferase reporter, as detailed in the experimental section. This assay, while dependent on both viral (L protein) and cellular polymerases, provides an indirect readout of the polymerase complex function. Using this approach, we observed inhibitory effects of the selected compounds, with IC_50_ = 5.9 ± 1.1 μM for 2′-FdC and IC_50_ = 1.6 ± 0.2 μM for EIDD-1931 (Figure S2).

### RVFV Develops Full Resistance to 2′-FdC and Partial Resistance to EIDD-1931

Based on our screening results, we performed serial passaging in the presence of 2′-FdC or EIDD-1931 to select decreased-susceptibility variants and map resistance-associated mutations of the ZH-548 strain of RVFV (r548-FdC and r548-EIDD) by treating the virus with increasing inhibitor concentrations. As a positive control and to determine any naturally occurring mutations, ZH-548 was also serially passaged without the presence of an inhibitor. As a negative control, VERO E6 cells were passaged without virus or an inhibitor. After the cytopathic effect was well-developed (90–100%) in the presence of the inhibitor, the virus-containing supernatant was harvested and used to infect the next passage. Interestingly, beginning at passage 7, propagation of ZH-548 in the presence of EIDD-1931 led to increases in the time period during which the cytopathic effect developed—from 3 to 5 days at passage 7 to 6 days at passage 8 and to 8 days at passage 9 (Figure 2). The 2′-FdC mutant fitness remained unchanged, as the cytopathic effect developed consistently over 3 days (Figures S4 and S5). When the last passage was reached, the resulting adapted viruses were rescreened against their respective inhibitors across a concentration range of 0.16–100 μM. r548-FdC developed seemingly complete resistance, with IC_50_ > 100 μM, while r548-EIDD variant developed partial resistance, with IC_50_ = 13 ± 2 μM.

**Figure 2:**
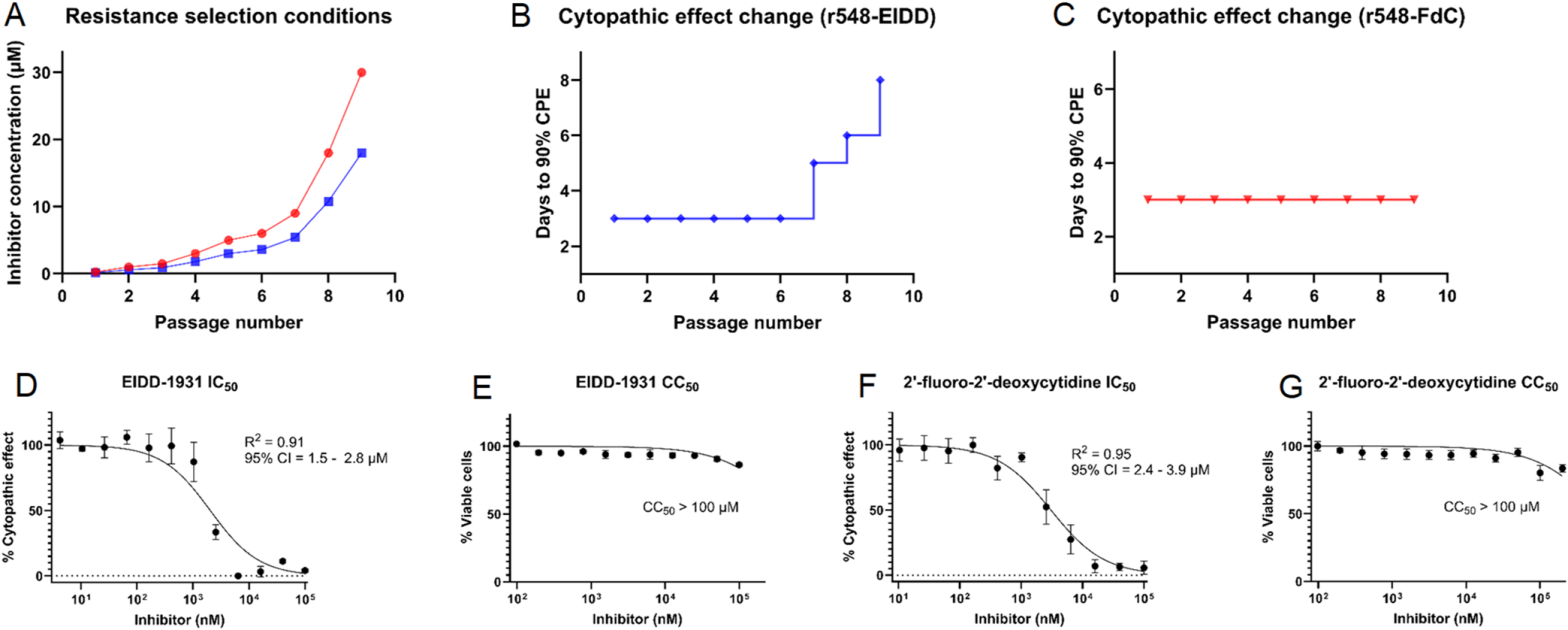
Conditions for the generation of resistant variants and observed cytopathic effect changes in the increasing presence of an inhibitor. RVFV (ZH-548 strain) was propagated in an increasing fraction of the IC_50_ concentrations (concentrations of 2′-FdC are denoted in red, EIDD-1931 in blue) measured during the inhibitor screening. (**A**) Virus-containing supernatant was harvested after the cytopathic effect was fully developed (>90% of cell culture). During the virus cultivation, formation of the cytopathic effect was greatly delayed for r548-EIDD (**C**), but not for r548-FdC (**B**), suggesting a propagation of partially and fully resistant variants, respectively. The antiviral activity of EIDD-1931 (**D**) and 2′-FdC (**F**) was determined using a cytopathic effect (CPE) reduction assay, with IC_50_ values measured as 2.0 ± 0.3 and 3.0 ± 0.3 μM, respectively. Cytotoxicity of EIDD-1931 (**E**) and 2′-FdC (**G**) was assessed in parallel, showing that both compounds are nontoxic in selected concentration range. Antiviral testing and cytotoxicity evaluation (D–G) were performed in biological triplicates, and titration curves were fitted using nonlinear regression as inhibitor concentration versus normalized response, with 95% confidence intervals calculated.

Following Sanger sequencing of the mutated virus variants, we obtained data about the selected mutations in all three genomic segments (Tables 1 and S1).

**Table 1:** Summary of the Nonsynonymous Mutations Revealed by Sequencing in All Three Genomic Segments of the RVFV after the 9th Passage

Segment	---	2′-FdC	EIDD-1931
L	D1506N	N76S	R43G
		G775R	L144F
		A1180S	V164I
		I1480V	M749I
		V2061I	S790L
			S1667N
M	–	Q286L	V66I
			G93S
			G289D
			A418V
S	–	–	NSs: V36I
			R47H
			V192I
			V195I
			V241I
			NP: K52R

The active form of molnupiravir (EIDD-1931) caused an accumulation of 6 nonsynonymous mutations and 20 synonymous mutations in the L segment of the virus coding for the L protein, 4 nonsynonymous mutations and 5 synonymous in the M segment coding for surface glycoproteins, and 6 nonsynonymous and 3 synonymous in the S segment coding for nucleoprotein and nonstructural protein. Given its mode of action, this result confirmed the role of molnupiravir as a powerful mutagenic agent.^38^

The mutations on the L segment are distributed across all three domains. The endonuclease domain (ENDO, residues 1–214) contains 3 nonsynonymous mutations, the RdRp (residues 215–1585) contains 2, and the C-terminal part containing the cap-binding domain (residues 1586–2092) contains 1 nonsynonymous mutation. On the other hand, 2′-FdC caused 5 nonsynonymous mutations and 3 synonymous mutations in the L segment. Out of these 5 nonsynonymous mutations, 1 is localized in the ENDO domain, 3 are in the RdRp domain, and 1 is in the C-terminal domain. One nonsynonymous and 1 synonymous mutation were localized in the M segment. The virus treated with 2′-FdC did not develop any substitutions in the S segment of its genome. The positive control (r548-no inhibitor) revealed a single mutation in the L segment of the virus (Figure 3). The mutations in the RVFV L segment induced by EIDD-1931 and 2′-FdC were subsequently structurally analyzed in detail.

**Figure 3:**
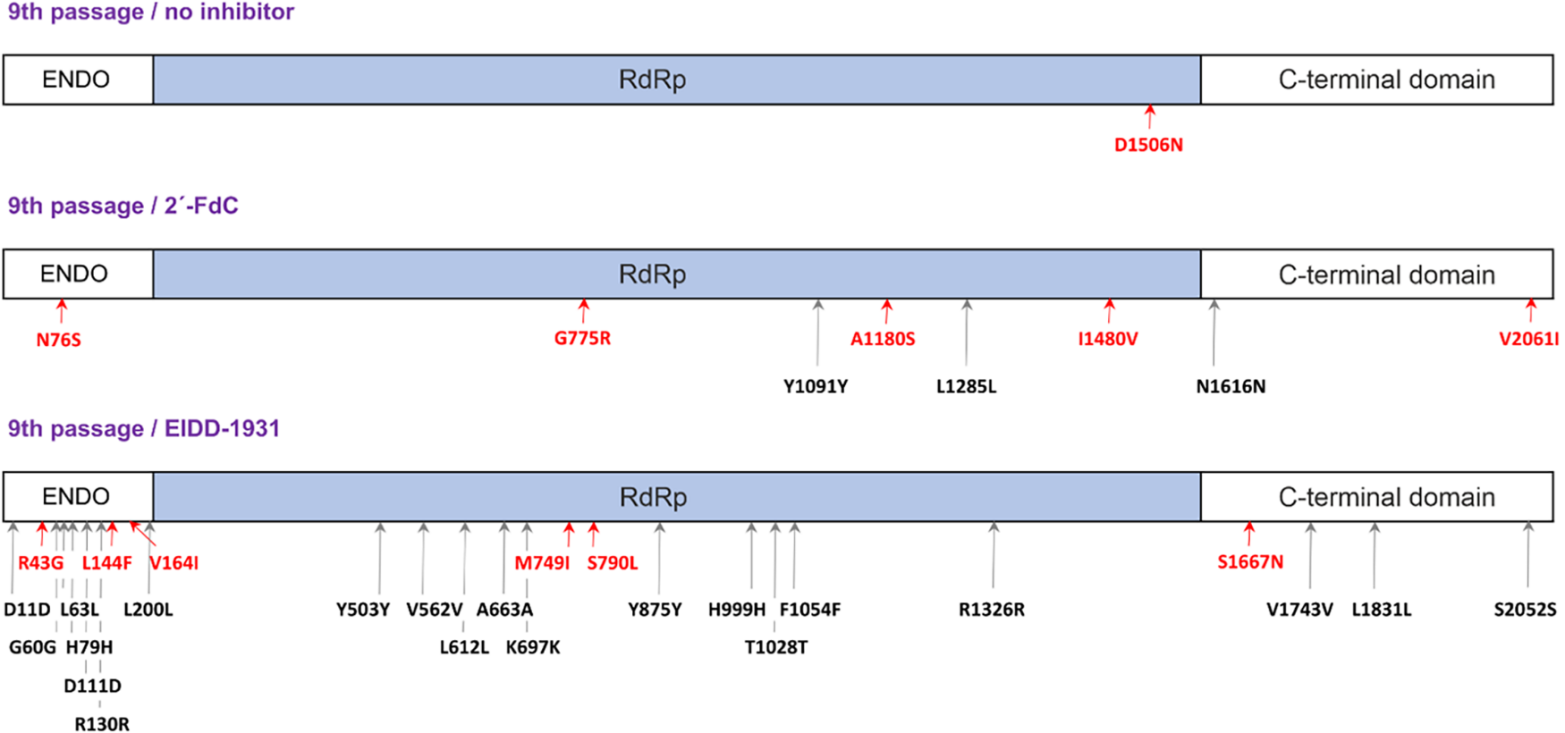
Visual representation of the mutations located in the L segment encoding the RVFV L protein. The nonsynonymous mutations are denoted in red and synonymous mutations in black. Both inhibitors caused mutations in all three domains of the L protein.

### L Protein_apo_ Structure Provides a Basis to Understand the Structural Impact of Resistance Mutations

We determined the cryo-EM structure of the RVFV L protein in the apo form at 3.5 Å (L protein_apo_), which resolves the RdRp while leaving several peripheral regions flexible (Figures S6 and S7). This apo structure aligns closely with the previously resolved RVFV L protein structure^31^ (RMSD ≈ 1.05 Å; Figure S7F), with positional differences located in the vRNA-binding lobe (secondary binding site and template exit channel) and in segments linked to dynamic domains (ENDO and C-terminal). The most notable difference is an ∼20 residue register shift starting near residue 1451. Both the RVFV AlphaFold prediction and the SFTSV (strain AH12) L protein structure^35^ indicate a loop in this region (Figure S7G), whereas the prior RVFV model^31^ omitted the loop and threaded the sequence into a neighboring helix, producing the ∼20-residue shift (Figure S7G,H). Although our density does not resolve this loop unambiguously, the downstream register aligns with the AlphaFold and the SFTSV models,^35^ supporting our revised assignment beyond this segment.

The RVFV L protein_apo_ shares a similar overall organization with the influenza virus polymerase subunits (PA, PB1, PB2)^39–41^ and other bunyavirus L proteins,^39,42,43^ reflecting their common evolutionary origin. The catalytic RdRp domain (resembling the PB1 domain) contains the conserved thumb, finger, and palm subdomains seen in SFTSV^27,35^ and La Crosse virus (LACV)^28,29^ L proteins (Figures 4A,B, S8A). In the RVFV L protein_apo_, the finger subdomain adopts a more open, inactive conformation than the replicating state observed for SFTSV^35^ (Figure S9A). The PA-like region also encompasses the vRBL subdomain, which provides viral RNA binding sites.^31,39^ Notably, the 5′ RNA hook–binding cleft is closed in the L protein_apo_ (preopened in SFTSV^30,36^ and LACV L proteins^29^), whereas the 3′ RNA secondary binding site is disordered (Figures 4A,B and S8B). The ENDO and C-terminal domains are highly flexible and unresolved compared to SFTSV and LACV L proteins (Figure S8C).^29,30,36^ Together, these features show that bunyaviral L proteins depend on RNA-induced conformational flexibility and structural rearrangements.

**Figure 4:**
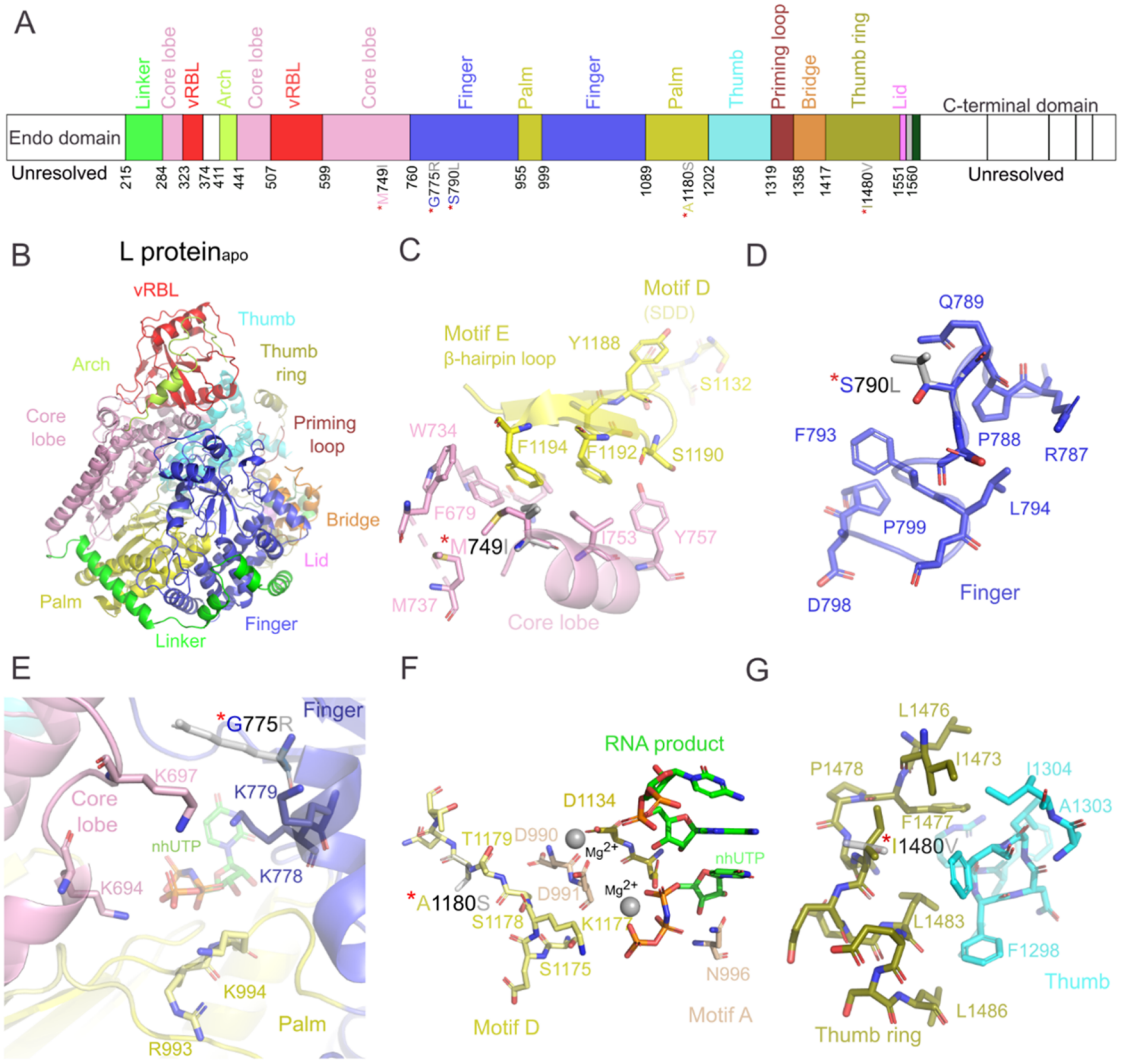
Cryo-EM structure of RVFV L protein_apo_ and localization of EIDD-1931 and 2′-FdC resistance mutations. (**A**) Schematic representation of the L protein’s structural elements mapped onto its amino acid sequence. (**B**) Cryo-EM structure of L protein_apo_ in cartoon representation, with domains and subdomains color-coded as in panel (**A**). (**C**) Structural model representation of the M749I mutation (core lobe region), highlighting the I749 mutant in gray. (**D**) Structural model representation of the S790L mutation (finger subdomain), highlighting the L790 mutant in gray. (**E**) Structural model representation of the G775R mutation (finger subdomain), highlighting the R775 mutant in gray. The position of nonhydrolyzable UTP analog (nhUTP) is adapted from the replicating SFTSV L protein (PDB 8ASD)^35^ based on main chain (Cα) superposition to L protein_apo_. (**F**) Structural model representation of the A1180S mutation (palm subdomain), highlighting the S1180 mutant in gray. The positions of nhUTP, Mg^2+^, and RNA product are adapted from the replicating SFTSV L protein (PDB 8ASD)^35^ based on main chain (Cα) superposition to L protein_apo_. (**G**) Structural model representation of the I1480V mutation (thumb ring), highlighting the V1480 mutant in gray.

The RVFV L protein_apo_ structure provided a framework to map resistance mutations selected by EIDD-1931 and 2′-FdC. For EIDD-1931, two substitutions (M749I and S790L) lie near the catalytic RdRp (Figures 4C,D and S10A), while additional substitutions appear in the flexible ENDO and C-terminal regions (Figure S10). M749 sits adjacent to motif E, which stabilizes the active site and contacts motif D during RNA synthesis.^44^ In this region, the motif E β-hairpin is stabilized by a hydrophobic pocket formed by residues from motif E (F1192, F1194) and the core lobe (M749, I753, Y757, W734, F679, and M737) (Figure 4C), including a critical sulfur−π interaction between M749 and F1194.^45^ Replacing methionine with isoleucine could weaken this pocket and may destabilize the β-hairpin, potentially explaining the mild resistance phenotype, although the presence of leucine at the equivalent position in SFTSV argues that the effect may be limited (Figures 4C and S10A). The S790L resistance mutation (conserved serine occupies the equivalent position in SFTSV) lies on a solvent-exposed helix in the finger subdomain (Figures 4D and S10A) and may affect local stability, but its broader impact on polymerase function remains unclear. EIDD-1931 selected mutations L144F and V164I in the ENDO domain (the equivalent positions are occupied by isoleucine and valine in SFTSV) (Figure S10A,C,D) sit near the coordination site (residues D111, E125, and K143) of two Mn^2+^ ions essential for cap-snatching.^7,34,46^ AlphaFold guided inspection of the RVFV ENDO domain, together with similar residue chemistry and geometry observed in the ENDO domain of SFTSV L protein^35^ and Toscana virus (TOSV),^47^ suggests that these mutations could reshape the pocket and alter the endonuclease and metal binding activity (Figure S10A,C,D). The structural analysis of the additional substitutions (R43G in ENDO; S1667N in the C-terminal region) did not reveal a clear mechanism for resistance (Figure S10A,B,E). Taken together, the modest increase in IC_50_ to EIDD-1931 suggests these substitutions do not confer strong direct resistance. Instead, they are mostly consistent with the mutagenic action of EIDD-1931 (G→A and C→U transitions),^38^ which lowers L protein fitness and yields a moderate, indirect resistance phenotype.

We further analyzed the structural impact of 2′-FdC resistance mutations on the RVFV L protein_apo_. First, we concentrated on the mutations G775R, A1180S, and I1480V which are located in the finger and palm subdomains, and thumb ring, respectively (Figures 4A–G and S11A). These mutations are proximal to the PB1-like RdRp core and can influence viral gene regulation via the conserved bunyaviral polymerization mechanism by altering nucleotide discrimination, RNA binding, and the fidelity of the RVFV L protein.^39,43^ The G775R mutation (the equivalent position is occupied by glutamate in SFTSV) (Figures 4E and S11A) can play a critical role in nucleotide entry by altering the electrostatic environment of the nucleotide entry channel. The channel is lined with positively charged residues from the finger (K778, K779), palm (K993, R994), and core lobe (K694, K697) subdomains, facilitating the selective entry of negatively charged NTPs into the active site (Figure 4E). Here, the G775R mutation introduces an additional positively charged arginine. Moreover, the bulky arginine side chain may sterically hinder 2′-FdC entry, preventing its incorporation and conferring resistance, underscoring the nucleotide entry channel’s role in RNA replication fidelity.^48^ In the elongating SFTSV L structure, the RVFV G775R mutation would be placed near the 5′ RNA hook region and the fingertip.^35^ This proximity suggests that the G775R mutation could contact the RNA backbone and potentially affect the 5′-hook binding and the fingertip-driven transition to the active conformation (Figure S11A,D). The A1180S mutation (the equivalent position is occupied by valine in SFTSV) in the palm subdomain lies within motif D, near the active site (Figures 4F and S11A). By the introduction of an additional serine, this mutation potentially enhances fidelity through altered electrostatics and hydrogen bonding. Motif D is a flexible element that undergoes large conformational changes to position itself near the β-phosphate of the incoming NTP, ensuring accurate selection and incorporation (Figure 4F).^48–50^ Motif D functions as a general acid during catalysis, with K1177 protonating the pyrophosphate.^50^ The deprotonation of K1177 is stabilized by hydroxyl interactions from nearby polar residues S1175, S1178, and T1179 within motif D (Figure 4F). The A1180S mutation may further stabilize these interactions, refining K1177 deprotonation and improving fidelity, unlike mutations that reduce fidelity by removing polar residues (e.g., T362I in poliovirus)^51^ and therefore enhance the discrimination against nucleotide analogs like 2′-FdC. The I1480V mutation (the equivalent position is occupied by alanine in SFTSV) is located within the conserved hydrophobic pocket of the thumb subdomain and thumb ring (Figures 4A,G and S11A). The thumb ring is essential for genome replication, facilitating the transition from initiation to elongation, guiding the template and nascent RNA strand separation and providing stability.^33,35^ Structural studies of related viruses, including SFTSV and LACV, reveal that interactions between the thumb ring and the template exit region undergo substantial conformational changes during elongation (Figure S9B).^28,35^ The I1480V mutation appears to modulate the fidelity of the L protein by coordinating effects across the replication process, thereby preventing the incorporation of 2′-FdC. This effect is likely mediated through stricter regulation of conformational transitions and altered interactions with the template exit region. A previously reported A1303T mutation in RVFV L protein, located within the same hydrophobic pocket, confers resistance to favipiravir by altering the polymerase fidelity, further supporting this mechanistic interpretation.^52^ The N76S and V2061I mutations are positioned in the flexible and unresolved ENDO and C-terminal domains, respectively. Structural analysis using an AlphaFold-predicted model of the RVFV ENDO domain, compared with the TOSV endonuclease structure,^47^ suggests that the N76S mutation in RVFV is located in close proximity to the metal-binding pocket, as defined by the catalytic mutant H78A in TOSV.^47^ This proximity suggests that the N76S mutation could influence the catalytic efficiency or overall activity of the RVFV L protein. In contrast, the structural analysis of the V2061I mutation in C-terminal domain did not reveal any plausible structural mechanism that could explain its association with resistance to 2′-FdC (Figure S11A–C).

### RVFV L Protein Undergoes Sequential Adaptation as It Develops Resistance against EIDD-1931 and 2′-FdC

To monitor resistance development to EIDD-1931 and 2′-FdC, we reevaluated inhibitor sensitivity across all passages. Resistance to EIDD-1931 appeared mild at passage 9, but high resistance to 2′-FdC emerged at passage 7. The viruses up to passage 8 (EIDD-1931) or passage 6 (2′-FdC) showed sensitivities similar to those of the wild-type virus (Figure 5). To pinpoint the sequence of the mutations responsible for the resistance, we isolated total viral RNA from all passages of viruses, transcribed the RNAs coding for L segment into cDNA, and sequenced them. This allowed us to track the emergence of resistance mutations over time.

**Figure 5:**
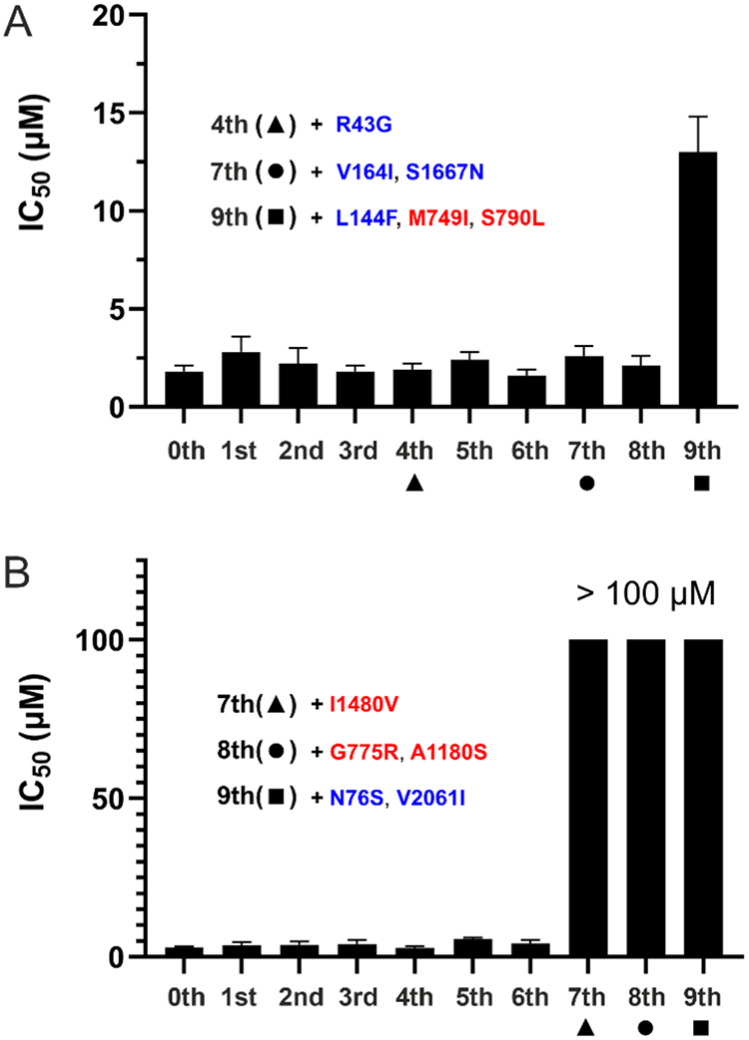
Development of resistance against EIDD-1931 (**A**) and 2′-FdC (**B**). Viruses from each passage were titrated with the respective inhibitor, and IC_50_ values were determined. Nonsynonymous mutations located in the RdRp domain are shown in red, while those in the ENDO and C-terminal domains are shown in blue.

EIDD-1931 caused the emergence of three nonsynonymous mutations in passages 4 and 7 which did not have any effect on the susceptibility of the virus toward this inhibitor. In the last passage, however, two nonsynonymous mutations (M749I and S790L) emerge (Figure 5A). In the case of the virus cultivated in the presence of EIDD-1931 (r548-EIDD), the mutations developed in passage 7 caused a decrease in the rate of viral growth, as the amount of viral RNA decreased approximately 3-fold compared to the wild-type virus (Figure S12A). Consequently, the last passage displayed the slowest viral growth kinetics measured by RT-qPCR, compared to the wild-type virus and previous passages. Viral RNA levels decreased 80-fold compared to the previous passage and nearly 270-fold compared to the wild-type virus in the same time frame. Therefore, these mutations located near the active RdRp core can alter the structural architecture of this region, which may explain the mild inhibitor resistance that was observed.

In the presence of 2′-FdC, the I1480V mutation in the thumb ring region emerges first (seventh passage) (Figure 5B), likely enhancing replication fidelity and providing a general resistance mechanism that increases viral fitness despite the presence of 2′-FdC. As adaptation progresses, RVFV acquires targeted mutations such as A1180S in motif D and G775R in the NTP entry tunnel, which block 2′-FdC incorporation and prevent its access to the catalytic site, respectively. For the r548-FdC virus, the I1480V mutation selected in passage 7 conferred apparent complete resistance to the inhibitor, while also increasing viral replication (Figure S12B). Viral RNA levels were higher than those of the wild-type virus, peaking at 3 days post infection (DPI), where they achieved a 3-fold increase over the wild-type. In contrast, the addition of G775R and A1180S mutations selected in passage 8 reduced viral replication back to the baseline level, with RNA levels comparable to the wild-type virus. Further accumulation of mutations—N76S and V2061I, located in the endonuclease domain and C-terminal domain, respectively—led to a marked decrease in replication efficiency. At 3 DPI, viral RNA levels were approximately 5-fold lower than the wild-type. Despite this, a strong cytopathic effect (CPE) was still observed at 3 DPI, indicating that reduced viral replication alone does not abolish CPE formation. This sequential adaptation in the RVFV L protein follows a stepwise adaptive mechanism under strong selective pressure, where early mutations improve overall viral fitness, and later mutations fine-tune resistance while minimizing fitness costs.^53^

## Conclusion

In this study, we investigated the RVFV L protein (RdRp) as a potential target for the development of antiviral therapeutics, identifying inhibitors that effectively suppress viral replication. Our screening of a diverse panel of polymerase inhibitors revealed that some established antiviral agents exhibited limited efficacy, while others, such as 2′-FdC and the active metabolite of molnupiravir (EIDD-1931), demonstrated potent antiviral activity against RVFV. We confirmed the mode of action of 2′-FdC as a chain terminator, while EIDD-1931 functions as a powerful mutagen. Through serial passaging experiments, we generated resistant viral variants and identified key mutations in the L protein that conferred reduced inhibitor susceptibility.

The cryo-EM structure of L protein_apo_ provided a structural framework to interpret the nature of these resistance mutations and their mechanistic impact on the RdRp function. The identified mutations, particularly those in the nucleotide entry channel, palm subdomain, and thumb ring region, underscore critical sites involved in polymerase adaptation under selective pressure.

These insights advance our understanding of the molecular basis of resistance and offer guidance for the development of next-generation polymerase inhibitors with improved potency and a lower propensity for resistance. Given the structural conservation of L proteins across bunyaviruses, our findings contribute to the broader antiviral research landscape, emphasizing the value of structural insights for the rational design of polymerase inhibitors. Future investigations should evaluate the *in vivo* efficacy of these inhibitors and assess their potential for clinical development.

## Experimental Section

### Chemicals

Antivirals and nucleoside analogs were sourced from various commercial suppliers for this study. Favipiravir (T-705) was obtained from Carbosynth Limited, while pimodivir (VX-787) was acquired from AdooQ Science. Remdesivir (GS-5734) was purchased from Aobious. The following nucleoside analogs were procured from Biosynth Carbosynth: 2-fluoro-2-deoxycytidine, cyclopentenyl-cytosine, 2-fluoro-2-deoxythymidine, N4-acetyl-2-fluoro-deoxycytidine, and N4-benzoyl-2-fluoro-deoxycytidine. Additional compounds, including 5-fluoro-2-deoxycytidine and cyclopentenyluracil, were sourced from Sigma-Aldrich.

MedChemExpress supplied cidofovir (GS-0504), dasabuvir (ABT-333), sofosbuvir (GS-7977), 2-deoxy-2,2-difluorocytidine (gemcitabine), 2-deoxy-2-fluoro-2-C-methylcytidine (PSI-1630), 2-fluoro-2-deoxyadenosine, 2-fluoro-2-deoxyguanosine, 2-fluoro-2-deoxyuridine, 5-aza-2-deoxycytidine (decitabine), 5-azacytidine (ladakamycin), baloxavir marboxil, cytosine arabinoside (cytarabine), EIDD-1931, emtricitabine (FTC), gemcitabine elaidate (CP-4126), LY-2334737, lamivudine (3TC), molnupiravir (EIDD-2801), stavudine (d4T), T-1105, tenofovir alafenamide (TAF), ribavirin (ICN-1229), and zidovudine (AZT). All compounds were used as received without further purification. The purity of all chemicals was confirmed to be >96% according to the Certificates of Analysis provided by the suppliers.

### Antiviral Activity Screening of Selected Inhibitors

RVFV strain ZH-548 was kindly provided by Dr. Frederic Arnaud of Claude-Bernard University, Lyon. Antiviral activity was measured by determining the extent to which the screened compounds reduced the virus-induced cytopathic effect (CPE). The day before infection, 2 × 10^4^ VERO E6 cells in a total volume of 100 μL were seeded in 96-well clear plates in DMEM complete medium (10% v/v FBS, 1% v/v penicillin/streptomycin) and allowed to attach overnight. Afterward, the medium was exchanged for 80 μL of virus growth medium (DMEM, 2% v/v FBS, 1% v/v Penicillin/Streptomycin), and 10 μL of test compound diluted in Opti-MEM (Gibco) was added to final concentrations of 100 and 25 μM (Figure S1A). Cells were incubated for 1 h at 37 °C, 5% CO_2_. Afterward, 10 μL of virus inoculum diluted in virus growth medium was added to the medium at MOI = 0.02. Following incubation at 37 °C in 5% CO_2_ for 3 days, cell viability was determined by the addition of 50 μL of XTT solution (Sigma-Aldrich). Cells were incubated for 4 h at 37 °C in 5% CO_2_, and the absorbance at 450 nm of newly formed orange formazan solution was measured using an EnVision plate reader (PerkinElmer, Waltham, USA). The resulting data were analyzed using GraphPad Prism 10.0 software (GraphPad Software, San Diego, CA, USA). Based on the results of the initial screening, selected hits (compounds inhibiting virus at one of the test concentrations) were rescreened using a dilution series of compounds to obtain titration curves. Titration curves were fitted using nonlinear regression as inhibitor concentration versus normalized response with 95% confidence intervals calculated (Figure S1B).

### Cytotoxicity Evaluation

The cytotoxic concentrations that reduced target cell viability by 50% (CC_50_) were determined by incubating serial dilutions of each tested compound with the selected cell cultures in the absence of the virus. VERO E6 cells were seeded at 2 × 10^3^ cells per well in 100 μL of DMEM complete (10% v/v FBS, 1% v/v Penicillin/Streptomycin) medium. The following day, the media were exchanged for the virus growth medium (DMEM, 2% v/v FBS, 1% v/v penicillin/streptomycin), tested compounds were added to the corresponding well, and the cells were incubated for 72 h at 37 °C in 5% CO_2_. After incubation, cell viability was analyzed by XTT colorimetric assay. 50 μL of 50:1 mixture of XTT labeling reagent (1 mg/mL) and PMS electron-coupling reagent (0.383 mg/mL) were added to the wells and incubated for 4 h at 37 °C in 5% CO_2_. Formation of orange formazan dye was measured in an EnVision plate reader (PerkinElmer, Waltham, USA). All experiments were performed in biological triplicate, and data were analyzed using GraphPad Prism 10.0 (GraphPad Software, San Diego, CA, USA). Where applicable, titration curves were fitted using nonlinear regression as inhibitor concentration versus normalized response, with 95% confidence intervals calculated (Figure S3).

### Minigenome Assay

VERO E6 cells (3 × 10^4^ per well) were seeded in 96-well plates in a total volume of 100 μL DMEM complete medium (10% v/v FBS, 1% v/v penicillin/streptomycin) and allowed to attach and grow overnight at 37 °C, 5% CO_2_. The following day, growth medium was exchanged for IMDM complete medium (10% v/v FBS, 1% v/v penicillin/streptomycin), and cells were treated by a dilution series of selected inhibitors in Opti-MEM (Gibco). Afterward, the cells were transfected with a mixture of plasmids comprising the active RVFV L protein (pCAGGS-L, pCAGGS-N), the firefly luciferase reporter gene (pPol1-ffRV), and β-d-galactosidase internal control gene (pcDNA3.1/His/LacZ). Briefly, a DNA mixture was prepared by mixing 80 ng per well of pCAGGS-L and pCAGGS-N, 40 ng per well of pPol1-ffRV, and 20 ng per well of pcDNA3.1/His/LacZ. The mixture was diluted to an appropriate volume with Opti-MEM (Gibco). The transfection mixture was prepared by diluting Lipofectamine-2000 in Opti-MEM (Gibco) in a 3:1 ratio proportional to the mass of the DNA and incubated for 20 min at room temperature. After the addition of the transfection mixture, cells were incubated for 48 h at 37 °C, 5% CO_2_. Following the incubation, the firefly luciferase activity was measured using the OneGlo kit (Promega) according to the manufacturer’s instructions. To measure β-d-galactosidase activity, 20 μL of cell lysate was transferred to a clear 96-well plate, and 90 μL of β-galactosidase substrate mixture was added (100 mM Na_2_HPO_4_, pH 7.0; 1 mM MgCl_2_; 45 mM β-mercaptoethanol; 4 mM o-nitrophenyl-β-d-galactopyranoside) to each well. The mixture was incubated for 1 h at 37 °C, 5% CO_2_. Afterward, 150 μL of 1 M NaHCO_3_ was added, and absorbance at 450 nm was measured using an EnVision plate reader (PerkinElmer, Waltham, USA). The experiments were performed in biological triplicates, and the resulting data were analyzed using GraphPad Prism 10.0 software (GraphPad Software, San Diego, CA, USA). Titration curves were fitted using nonlinear regression as inhibitor concentration versus normalized response with 95% confidence intervals calculated (Figure S2).

### Selection of Resistant Variants

To generate mutants under the selection pressure of inhibitors, we opted for the serial passaging of the virus in the presence of increasing concentrations of the compound. Selections were conducted under institutional approval and with appropriate biosafety containment. The procedure did not involve host-range alteration or manipulations intended to increase virulence; it was limited to drug-pressure selection in a cell culture to identify on-target resistance and associated fitness costs. The day before infection, 1.5 × 10^6^ VERO E6 cells were seeded in 25 cm^2^ flasks in DMEM complete medium (10% v/v FBS, 1% v/v Penicillin/Streptomycin) and left to incubate overnight at 37 °C in a 5% CO_2_ incubator. DMEM complete medium was then exchanged for virus growth medium (DMEM; 2% v/v FBS, 1% v/v penicillin/streptomycin), and inhibitor diluted to the selected concentration was added, starting at 0.1× IC_50_ and ending at 10× IC_50_ at the ninth and final passage. Cells were incubated for 1 h at 37 °C, 5% CO_2_, and then infected with RVFV strain ZH-548 at MOI = 0.02. Cells were monitored for the formation of CPE, and after the indicated number of days, the supernatant from the flask was harvested, centrifuged at 4000*g*, 4 °C, for 10 min, filtered through a sterile 0.45 μm filter (Carl Roth GmbH), aliquoted, and stored at −80 °C. A 100 μL aliquot of the supernatant was then used to reinfect the next passage with increased inhibitor concentration. As a negative control, uninfected VERO E6 cells were propagated in the virus growth media, and as an internal control, virus was passaged without the presence of an inhibitor.

### Viral RNA Isolation, Transcription, and Amplification

Total viral RNA was isolated from the supernatant of the infected cell cultures after CPE was observed. Isolation was performed using the QIAamp Viral RNA Kit (Qiagen) according to the manufacturer’s instructions. Isolated viral RNA was transcribed into cDNA using the SuperScript IV First-Strand Synthesis System via the primers listed in Table S2. The L segment of the viral genomic RNA was transcribed and amplified in two overlapping amplicons due to its size. This approach was also used for the S segment due to the presence of the hairpin secondary structure, which hindered transcription into a single amplicon. The M segment was amplified with a single amplicon. The obtained cDNAs were then amplified by Phusion High-Fidelity DNA Polymerase (New England Biolabs) using the same primers as in the reverse transcription step. The resultant DNA was visualized on a 1% agarose gel (Lachmann) stained with GelRed Nucleic Acid Gel Stain (Sigma-Aldrich). DNA bands of sizes corresponding to the amplified viral RNA were cut out with sterile surgical scalpels and isolated using the QIAquick Gel Extraction Kit (Qiagen) according to the manufacturer’s instructions. Isolated DNA was analyzed via Sanger sequencing using the set of primers listed in Table S2. The electropherograms are shown in Figures S13 and S14, and quantitative metrics are shown in Table S3. Mutated sites were considered in cases where the height of the overlapping peak in Sanger sequencing reached >15% of dominant variant while, at the same time, the signal-to-noise ratio in 6 surrounding nucleotides did not subside 99%. Mutations detected based on these criteria are listed in Table S1.

### Virus Growth Kinetics of Resistant Variants

To describe how the mutations in the viral genome influence the growth kinetics of the virus, we employed an RT-PCR strategy. Viral RNA was quantified from cell culture supernatants collected at 1, 2, and 3 days post infection (DPI) from cells treated with increasing concentrations of the inhibitor. Supernatants were inactivated by heating at 85 °C for 15 min to ensure biosafety. RNA detection was performed using the Luna Universal One-Step RT-qPCR Kit (New England Biolabs, Ipswich, MA, USA) according to the manufacturer’s protocol. A pair of primers targeting a 150 bp fragment of the L segment of Rift Valley fever virus (RVFV) were used: forward primer (LF2) and reverse primer (LQRT) (Table S2). Reactions were carried out in a total volume of 20 μL containing 5 μL of heat-inactivated supernatant, with amplification performed on a CFX96 real-time system (BioRad). All samples were run in technical duplicates, and results were expressed as fold change in viral RNA levels relative to baseline, plotted against days post infection.

### Expression and Purification of Full-Length Wild-Type L Protein

The Bac-to-Bac method (Thermo Fisher Scientific) was used to recombinantly express the L protein. The coding sequence for the RVFV L-gene (GenBank accession number DQ375403.1) featuring a Twin-Strep-tag at the C-terminus and a D103A mutation, which enhances protein expression,^31^ was cloned into the pFastBac1 vector and transformed into DH10-YFT *Escherichia coli*. Following the transformation, white colonies were inoculated into 10 mL of LB media and incubated overnight, and the resulting bacmid DNA was isolated. To obtain recombinant baculovirus, Sf9 cells were transfected with the bacmid using an ExpiFectamine-Sf transfection reagent. Following the formation and amplification of the recombinant baculovirus, expression of wild-type L protein was confirmed by Western blot using StrepMAB-Classic antibodies (IBA Lifesciences). For expression of Twin-Strep-tagged L protein, the Sf9 cells were infected at 2 × 10^6^ cells/ml with 0.1% v/v of recombinant baculovirus. The infected Sf9 cells were incubated at 27 °C, 200 rpm, in SFM-900 II (Gibco) medium and harvested between 96 and 120 h after infection. The cell suspension was centrifuged at 500*g* for 5 min, and pellets were resuspended in Buffer A (100 mM Tris-HCl, pH 8.0, 150 mM NaCl) with cOmplete protease inhibitor cocktail (Roche).

The cells were lysed by sonication with a SonoPlus UW 2200 instrument (Bandelin electronic GmbH&Co) followed by centrifugation 30,000*g*, for 2 h at 4 °C. Soluble L protein was purified on Strep-Tactin XT 4Flow resin (IBA Lifesciences) and eluted with elution buffer (100 mM Tris-HCl, pH 8.0, 150 mM NaCl, 5% v/v glycerol, 2 mM TCEP, 1 mM EDTA, 50 mM biotin). Eluted fractions were dialyzed in Buffer B (100 mM Tris-HCl, pH 8.0, 150 mM NaCl, 5% v/v glycerol, 2 mM TCEP) overnight. Dialyzed L protein was further loaded onto a MonoQ column (HiTrap Q HP, Cytiva) and eluted with a gradual gradient of Buffer C (100 mM Tris-HCl, pH 8.0, 2 M NaCl, 5% v/v glycerol, 2 mM TCEP). Based on SDS-PAGE, pure fractions of wild-type L protein were pulled together and concentrated using ultra centrifugal filters (Amicon Ultra 10K, Merck Millipore). Concentrated L protein was flash frozen and stored at −80 °C until further use.

### Cryo-EM Grid Preparation

A 3.5 μL droplet of the apo form of wild-type RVFV L protein (2 μM) in buffer (100 mM Tris-HCl, pH 8, 250 mM NaCl, 2 mM DTT) was applied to a Quantifoil R 1.2/1.3 Cu 300 mesh grid. Prior to application, the grid was glow-discharged for 45 s at 40 W power and 5 W range using a Gatan Solaris II. After sample application, grids were blotted for 5 s with zero blot force and plunge frozen in liquid ethane cooled by liquid nitrogen using an FEI Vitrobot Mark IV at 4 °C and 100% humidity.

### Cryo-EM Data Collection and Processing

A total of 9046 micrographs of the RVFV L protein were collected on a Titan Krios microscope (Thermo Fisher Scientific) operating at 300 kV, equipped with a K3 direct electron detector and a BioQuantum K3 Imaging filter (10 eV slit width, Gatan Inc.). Data acquisition was performed using SerialEM^54^ with a defocus range of −1.3 to −2.5 μm. Each exposure was captured as a 40-frame movie with a total dose of 40 e^–^/Å^2^ at a nominal magnification of 105,000×, corresponding to a pixel size of 0.8336 Å. Motion correction and frame averaging were performed using MotionCorr2,^55^ and further processing, including CTF estimation and correction (CTFFIND4),^56^ was conducted in CryoSPARC^57^ (Figure S6). A total of 596 micrographs were excluded due to beam-induced motion, excessive ice thickness, or contamination.

Initial particle picking was performed using a blob-based approach, followed by template-based particle picking, resulting in 2,403,715 particles. These particles were subjected to 2D classification into 50 classes by using a box size of 256 pixels. Five well-defined classes were selected based on sharpness and structural details, generating a particle stack yielding 470,838 particles. All cryoSPARC^57^ processing was performed without binning, using original 1 × bin particle stacks. The selected particles were used to generate four ab initio 3D reconstructions, which were further refined and classified through 3D heterogeneous refinement into four classes using the ab initio maps as input templates. Two of the resulting classes were classified as junk, one corresponded to a low-resolution class, and one represented a class of L protein_apo_, resolved to an estimated resolution of 4.43 Å. This class contained 224,938 particles and was selected for further processing. First, the local or global CTF refinement of this particle stack did not improve the resolution of the L protein. Therefore, it was directly subjected to the next round of 3D heterogeneous refinement, carried out across three classes, to enhance particle classification and further resolve the structural details of L protein_apo_. The 3 volumes from the previous 3D heterogeneous refinement (representing classes 1–3) were used in this step as input template volumes. The process resulted in two low-resolution classes and one class of L protein_apo_ at an estimated resolution of 4.03 Å, containing 185,424 particles. The particle stack from the final selected class (185,424 particles) was then subjected to homogeneous refinement into a single class to enforce global alignment and enhance the resolution. This step improved the overall resolution of the map to 3.57 Å (FSC = 0.143). To further refine the resolution and structural details, local refinement was applied to the final class from homogeneous refinement, focusing on local flexibility. As a result, the final map (L protein_apo_) reached an overall resolution of 3.5 Å (FSC = 0.143). Additional 3D masking classifications with soft masks placed on the ENDO and C-terminal domain did not reveal any further well-resolved classes. Instead, the high flexibility of the domains further reduced the resolution of the L protein core. All refinement steps were carried out using the default settings in cryoSPARC.^57^ The final map was used for model building and structure refinements. Local-resolution filtering was applied to the resulting cryo-EM map (Figure S7A), using blocres and blocfilt from the Bsoft package (vs 1.9.1).^58^ The sharpening of the resulting cryo-EM map was performed with bfactor.exe^59^ using a constant B-factor of −160 Å^2^. Fourier shell correlation (FSC) curves were calculated using CryoSPARC^57^ (Figure S7B).

### Model Building and Refinement

The AlphaFold (AF)^60^ generated structure, a previous cryo-EM structure of the RVFV L protein (PDB 7EEI)^31^ and SFTSV L protein structure (PDB 8AS7)^35^ were used as starting models for structure refinement. The ENDO and C-terminal domains were omitted from the AF generated model, as they were not resolved or were very poorly defined in the cryo-EM map. The model was initially fitted to the cryo-EM map using rigid body fitting in Chimera,^61^ followed by manual adjustments in Coot.^62^ Highly flexible and unresolved regions, such as loops, were also omitted.

Structural refinement of L protein_apo_ was performed using phenix.real_space_refine in Phenix,^63^ with secondary structure restraints applied throughout the process to maintain proper geometry. Correlation coefficients (model-to-map fit) were carefully monitored to prevent overfitting of the model to the map. The refined structural model showed excellent alignment with the corresponding map, as indicated by the high correlation coefficients. FSC values between the final model and map were computed using Phenix (mtriage package),^63^ demonstrating strong agreement between the structural model and cryo-EM map (Figure S7C). The resulting model exhibited favorable stereochemical parameters, including minimal deviations from ideal bond lengths and angles and a low number of backbone outliers, as outlined in Table S4. Structure quality was evaluated using MolProbity.^64^ Structural superpositions and figure generation were performed using ChimeraX^65^ and PyMOL (The PyMOL Molecular Graphics System, Version 2.0 Schrödinger, LLC).

## Supplementary Material



## Notes

Nucleotide changes observed in the L, M, and S segments; list of primer sequences; quantitative metrics from Sanger sequencing; refinement statistics for the cryo-EM structure; CPE, CC_50_, and minireplicon titration curves; cytopathic effect documentation; cryo-EM data classification; global and local resolution of the structure; comparison with SFTSV and LACV L proteins; structural changes between RVFV and SFTSV L proteins in replication state; structural analysis of mutations in ENDO and C-terminal domains; growth kinetics curves; and Sanger sequencing analysis (PDF)

## Abbreviations

2′-FdC: 2′-fluoro-2′-deoxycytidine
CCHFV: Crimean-Congo hemorrhagic fever virus
CPE: cytopathic effect
cryo-EM: cryo-electron microscopy
DPI: days post infection
ENDO: endonuclease domain
FDA: Food and Drug Administration
HCV: hepatitis C virus
HIV: human immunodeficiency virus
LASV: Lassa virus
NP: nucleoprotein
NSs: nonstructural protein
RdRp: RNA-dependent RNA polymerase
RVFV: Rift Valley fever virus
SARS-CoV-2: severe acute respiratory syndrome coronavirus 2
SFTSV: severe fever with thrombocytopenia syndrome virus
TOSV: Toscana virus
XTT: cell proliferation assay
