# Supplementary material for: Targeting the Rift Valley Fever Virus Polymerase: Resistance Mechanisms and Structural Insights
Source: ACS Infect Dis. 2025 Oct 30;11(11):3364–76. doi: 10.1021/acsinfecdis.5c00832 (PMC12624712; doi:10.1021/acsinfecdis.5c00832)
Supplement: Supplementary file 1 [file id5c00832_si_001.pdf]

Supporting Information

Targeting the Rift Valley Fever Virus Polymerase: Resistance Mechanisms and Structural Insights

Michal Kráľ[1,2], Amiyaranjan Das[3,4], Tomáš Kotačka[1,2], Anna Blahošová[1,5], Veronika Liščáková[1,2], Jan Hodek[1], Jan Konvalinka[1, 6], Gabriel Demo[3*], Milan Kožíšek[1*]

1. Institute of Organic Chemistry and Biochemistry of the Czech Academy of Sciences, Flemingovo n. 2, 166 10, Prague 6, Czech Republic

2. First Faculty of Medicine, Charles University, Kateřinská 1660, 121 08, Prague 2, Czech Republic

3. Central European Institute of Technology, Masaryk University, Kamenice 753/5, 625 00, Brno, Czech Republic

4. National Centre for Biomolecular Research, Faculty of Science, Masaryk University, Kamenice 5, 625 00, Brno, Czech Republic

5. Department of Biology, Faculty of Science, Charles University, Viničná 7, 128 00, Prague 2, Czech Republic

6. Department of Biochemistry, Faculty of Science, Charles University, Hlavova 8, 128 00, Prague 2, Czech Republic

Corresponding Authors

* Milan Kožíšek, Institute of Organic Chemistry and Biochemistry of the Czech Academy of Sciences, Flemingovo n. 2, 166 10 Prague 6, Czech Republic
Tel: +420 220 183 518; e-mail: milan.kozisek@uochb.cas.cz
* Gabriel Demo, Central European Institute of Technology, Masaryk University, Kamenice 753/5, 625 00, Brno, Czech Republic
Tel: +420 549 497 815; e-mail: gabriel.demo@ceitec.muni.cz

Table S1. Overview of nucleotide changes observed in the L, M, and S segments of the RVFV genome after nine passages under compound selection pressure. The table summarizes the point mutations identified by Sanger sequencing across all three segments (L, M, and S) of the viral genome. First column represents genomic variants detected in the wild-type virus after nine passages in the absence of any selective pressure. The subsequent columns show mutations detected in viruses passaged in the presence of the nucleoside analogs 2´-FdC and EIDD-1931, respectively. Nucleotide substitutions are annotated according to HGMD nomenclature (https://www.hgmd.cf.ac.uk/docs/mut_nom.html). Non-synonymous mutations (that might potentially affect protein functions) are highlighted in red, while synonymous mutations not altering amino acid sequences are shown in black. Mutations are listed according to RVFV genomic segment (L, M, or S) and ordered by nucleotide position.

**----**		**2´- FdC**		**EIDD-1931**			
**L segment**							
D1506N	c.4534 G>A	N76S	c.245 A>G	D11D	c.51 C>T	K697K	c.2109 G>A
		G775R	c.2341 G>A	R43G	c.145 A>G	M749I	c.2265 G>A
		Y1091Y	c.3291 T>C	G60G	c.198 C>T	S790L	c.2387 C>T
		A1180S	c.3556 G>T	L63L	c.207 G>A	Y875Y	c.2643 C>T
		L1285L	c.3873 T>C	H79H	c.255 C>T	H999H	c.3015 T>C
		I1480V	c.4456 A>G	D111D	c.351 C>T	T1028T	c.3102 C>T
		N1616N	c.4866 T>C	R130R	c.408 G>A	F1054F	c.3180 T>C
		V2061I	c.6199 G>A	L144F	c.448 C>T	R1326R	c.3996 G>A
				V164I	c.508 G>A	S1667N	c.5018 G>A
				L200L	c.616 C>T	V1743V	c.5247 C>T
				Y503Y	c.1527 C>T	L1831L	c.5509 C>T
				V562V	c.1704 G>A	L1831L	c.5511 G>A
				L612L	c.1852 T>C	S2052S	c.6174 C>T
				A663A	c.2007 A>G		
**M segment**							
-		Q286L	c.877 A>T	V66I	c.216 G>A	C479C	c.1457 C>T
				G93S	c.297 G>A	G538G	c.1634 G>A
				T219T	c.677 C>T	G910G	c.2750 C>T
				G289D	c.886 G>A	R1123R	c.3389 G>A
				A418V	c.1273 C>T		
**S segment**							
-		-		**NS_s**		**NP**	
				V36I	c.140 G>A	R18R	c.1599 G>A
				R47H	c.174 G>A	K52R	c.1498 T>C
				L71L	c.247 T>C		
				C149C	c.481 C>T		
				V192I	c.608 G>A		
				V195I	c.617 G>A		
				V241I	c.755 G>A		

Table S2. List of primer sequences used for RNA transcription and Sanger sequencing.

Segment	Primer usage		Primer	Sequence (5' → 3')
L	RT-PCR	Amplicon 1	LcDNA-F	ACACAAAGGCGCCCAATCAT GGATTCTATATTATCAAAAC
			RVFL-3482b	CCACTTTGCATCTGGTAAGA ACCTTCTCATC
		Amplicon 2	RVFL_2845b	CATAGGGAAGTTCTTTGCTT CTGATACCCTC
			L_RT	ACACAAAGACCGCCCAATAT TGTAGCACTATGCTAGTATC
	Sequencing		LF1	ACACAAAGGCGCCCAATCAT
			LF2	CCTCCCTGGCTGTCCTATCA
			LF3	AAGCAGCAACTGAAGAGTTA
			LF4	GTGGAACCAAGGCCATTTTG
			LF5	TGGGCAGATGAAGATGTCAC
			LF6	TAATCTACAGAGTCCTAAGC
			LF6b	GGTTAGTAAGATAGCTATGGTTATC
			LF7	CGAGATGGAGTCTTTTGATA
			Eval1	GGTTTTGTTAGAGTGCCAATCAAGCATTTTG
			Eval2	TGAGCCCTTCATTCTTAAGAAG
			Eval3	GATGATAGTAGTATGCTAATCAG
			Eval4	CGTCCTGAGTTCAACTTCTTG
			Eval5	GTTCATTATCTATGATTGCTCATC
	Virus growth kinetics		LF2	CCTCCCTGGCTGTCCTATCA
			LQRT	ATCATCGTGCATCCTCTCAAT
M	RT-PCR		M_RT	ACACAAAGACCGGTGCAACT TCAAAGAGTTAGTTTAATTC
			MF1	ACACAAAGACGGTGCATTAA
	Sequencing		MF1	ACACAAAGACGGTGCATTAA
			MF2	AAGCACTCAAAAAGTGTGAT
			MF3	AGGGAACCCTGCCCCTATTC
			MF4	AAAGGAAATAGAGGTGTTCA
			MF5	GATTCTCCTCAGTCTTAAGGTCAAG
			MF6	CCTTTGCAATGATGGTGTCGTGAAC
S	RT-PCR	Amplicon 1	NF_F	ACACAAAGACCCCCTAGTGCT TATCAAGTATATCAT
			N3_R	GTGGGGCAGCCTTAACCTCTA
		Amplicon 2	N3_F	AACCCCCTGGGCAGCCACTTA
			N_RT	ACACAAAGCTCCCTAGAGATAC AAACACTATTACA
	Sequencing		NF0	TCTCTGAAAAGGCTTCGCTGGTGGAGG
			NF1	ACACAAAGACCCCCTAGTGC
			N3_F	AACCCCCTGGGCAGCCACTTA
			NF3	CATGGTGGATCCTTCTCTACCAGG

Table S3. Quantitative metrics from Sanger sequencing analysis of the viral L-segment for passage 9. The table presents key sequencing quality indicators and dominant mutation metrics derived from Sanger electrophoretograms of viral RNA collected after serial passaging. Metrics include read length, average Phred quality scores, number and type of nucleotide substitutions, and confirmation of dominant mutations. These data provide a quantitative summary supporting the progressive accumulation of mutations as observed in **Figure S13** and **S14**.

9th passage – EIDD-1931

Mutation (protein level)	**R43G**	**L144F**	**V164I**
Mutation (nucleotide level)	c.145A>G	c.448C>T	c.508G>A
Amplicon	ZH548_3482b_EIDD_LcDNAF	ZH548_3482b_EIDD_LcDNAF	ZH548_3482b_EIDD_LcDNAF
Position in amplicon	107	410	470
Basecalling QV	13	34	17
reference variant / peak height	432	488	330
mutation / peak height	166	132	224
mutation : genomic reference ratio	0,38	0,27	0,68
Trace Score	53	53	53
Contiguous Read Length	1082	1082	1082
QV20+	1071	1071	1071
Signal Intensity (A)	559	559	559
Signal Intensity (C)	548	548	548
Signal Intensity (G)	607	607	607
Signal Intensity (T)	921	921	921
basecaller	KB.bcp v3.0	KB.bcp v3.0	KB.bcp v3.0
mobility file	KB_3730_POP7_BDTv3.mob	KB_3730_POP7_BDTv3.mob	KB_3730_POP7_BDTv3.mob
instrument	ABI3730-18128-009	ABI3730-18128-009	ABI3730-18128-009
spacing	16,33	16,33	16,33

Mutation (protein level)	**M749I**	**S790L**	**S1667N**
Mutation (nucleotide level)	c.2265G>A	c.2387C>T	c.5018G>A
Amplicon	ZH548_3482b_EIDD_LF3	3482_EIDD_LF3	ZH548_2845b_EIDD_LF6b
Position in amplicon	238	361	158
Basecalling QV	17	51	14
reference variant / peak height	365	629	393
mutation / peak height	225	23	290
mutation : genomic reference ratio	0,62	0,04	0,74
Trace Score	52	51	55
Contiguous Read Length	1064	1050	1097
QV20+	1056	1063	1092
Signal Intensity (A)	640	116	742
Signal Intensity (C)	651	97	637
Signal Intensity (G)	689	174	855
Signal Intensity (T)	943	135	919
basecaller	KB.bcp v3.0	KB.bcp v3.0	KB.bcp v3.0
mobility file	KB_3730_POP7_BDTv3.mob	KB_3730_POP7_BDTv3.mob	KB_3730_POP7_BDTv3.mob
instrument	ABI3730-18128-009	ABI3730-18128-009	ABI3730-18128-009
spacing	16,08	15,54	15,99

9th passage – 2´-FdC

Mutation (protein level)	**N76S**	**G775R**	**A1180S**	**I1480V**	**V2061I**
Mutation (nucleotide level)	c.245A>G	c.2341G>A	c.3556G>T	c.4456A>G	c.6199G>A
Amplicon	ZH548_3482b_FdC_9th_LcDNAF	ZH548_3482b_FdC_9th_LF3	ZH548_2845b_FdC_9th_LF4	ZH548_2845b_FdC_9th_LF5	ZH548_2845b_FdC_9th_LF7
Position in amplicon	207	317	529	433	170
Basecalling QV	28	34	16	17	4
reference variant / peak height	A / 238	G / 627	G / 281	A / 422	G / 1718
mutation / peak height	G / 80	A / 134	T / 260	G / 389	A / 495
mutation : genomic reference ratio	0,34	0,21	0,93	0,92	0,29
Trace Score	52	54	54	54	47
Contiguous Read Length	1084	1090	1088	1081	339
QV20+	1068	1082	1080	1078	333
Signal Intensity (A)	481	439	423	309	388
Signal Intensity (C)	458	412	398	293	279
Signal Intensity (G)	710	608	673	430	566
Signal Intensity (T)	713	582	589	401	476
basecaller	KB.bcp v3.0	KB.bcp v3.0	KB.bcp v3.0	KB.bcp v3.0	KB.bcp v3.0
mobility file	KB_3730_POP7_BDTv3.mob	KB_3730_POP7_BDTv3.mob	KB_3730_POP7_BDTv3.mob	KB_3730_POP7_BDTv3.mob	KB_3730_POP7_BDTv3.mob
instrument	ABI3730-18128-009	ABI3730-18128-009	ABI3730-18128-009	ABI3730-18128-009	ABI3730-18128-009
spacing	16,13	16,19	16,07	15,98	16

Table S4. Refinement statistics for the cryo-EM structure of the L $protein_{apo}$

	L $protein_{apo}$
**PDBID**	9QTB
**EMDB**	EMD-53349
**Data collection and processing**	
Magnification	105,000x
Voltage (kV)	300
Electron exposure (e–/Å^2)	40
Defocus range (μm)	-1.3-2.5
Pixel size (Å)	0.8336
Symmetry imposed	C1
Initial particle (no.)	470,838
Final particle (no.)	185,424
Map resolution (Å)**	3.5
FSC threshold	0.143
**Refinement**	
Initial model used (PDB code)	7EEI
Correlation Coefficient (cc_mask)*	0.85
Map sharpening *B* factor (Å^2)	-160
Model composition*	
Non-hydrogen atoms	10304
Protein residues	1285
B factors (Å^2)*	
Protein	234.06
R.m.s. deviations#,$	
Bond lengths (Å)	0.004
Bond angles (°)	0.99
Validation#	
MolProbity score	1.54
Clashscore	4.76
Poor rotamers (%)	0
Ramachandran plot#	
Favored (%)	95.74
Allowed (%)	4.18
Disallowed (%)	0.08

** from CryoSPARC
* from Phenix
# from Molprobity
$ root mean square deviations

A

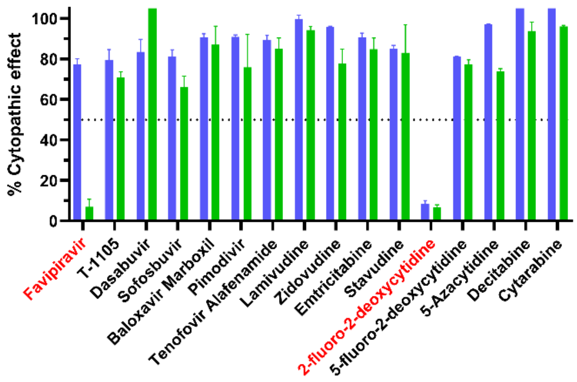

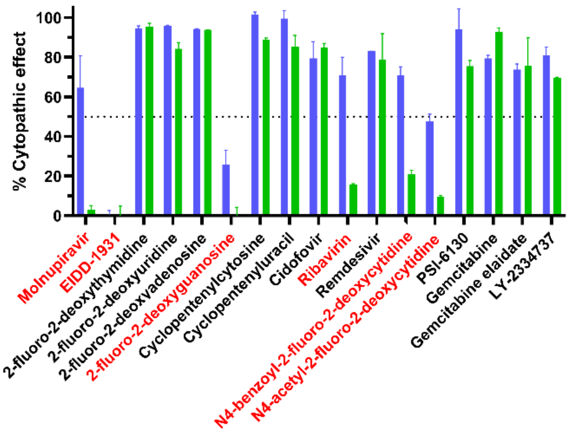

	Favipiravir		FTC (Emtricitabine)	
100 µM	2.511	2.404	1.036	1.179
25 µM	1.062	0.983	0.972	1.024
	T-1105		D4T (Stavudine)	
100 µM	1.191	1.112	1.318	0.96
25 µM	1.056	0.905	1.126	1.081
	Dasabuvir		2-fluoro-2-deoxyctidine	
100 µM	0.31	0.286	2.447	2.403
25 µM	0.996	0.841	2.444	2.482
	Sofosbuvir		5-fluoro-2-deoxycytidine	
100 µM	1.29	1.156	1.316	1.253
25 µM	0.918	1.001	1.218	1.208
	Baloxavir marboxil		5-Azacytidine	
100 µM	0.971	0.743	1.284	1.275
25 µM	0.811	0.77	0.607	0.612
	VX-787		Decitabine	
100 µM	0.802	0.776	0.697	0.585
25 µM	1.095	0.955	0.429	0.427
	Tenofovir		Cytarabine	
100 µM	0.962	0.827	0.609	0.593
25 µM	0.846	0.788	0.416	0.438
	Lamivudine		No virus	No drug
100 µM	0.942	0.99	2.659	0.839
25 µM	0.841	0.898	2.658	0.812
	AZT (Zidovudine)		No virus	No drug
100 µM	1.325	1.145	2.672	0.896
25 µM	0.908	0.901	2.77	0.817

	Molnupiravir		Cidofovir	
100 µM	1.426	1.003	0.916	0.868
25 µM	2.316	2.375	1.098	0.886
	EIDD-1931		Ribavirin	
100 µM	2.485	2.34	2.293	2.275
25 µM	2.363	2.423	1.281	1.021
	2-fluoro-2-deoxythymidine		Remdesivir	
100 µM	0.625	0.671	1.162	0.838
25 µM	0.683	0.646	0.93	0.927
	2-fluoro-2-deoxyuracil		N4-benzoyl-2-FdC	
100 µM	1.159	1.077	2.143	2.2
25 µM	0.901	0.909	1.222	1.092
	2-fluoro-2-deoxyadenosine		N4-acetyl-2-FdC	
100 µM	0.675	0.676	2.397	2.418
25 µM	0.671	0.666	1.577	1.688
	2-fluoro-2-deoxyguanosine		PSI-6130	
100 µM	2.365	2.479	1.019	1.103
25 µM	1.846	2.044	0.836	0.531
	Cyclopentenylcytosine		Gemcitabine	
100 µM	0.721	0.748	0.738	0.678
25 µM	0.485	0.516	0.957	1.003
	Cyclopentenyluracil		Gemcitabine elaidate	
100 µM	0.736	0.865	1.256	0.852
25 µM	0.528	0.618	1.138	1.054
	LY-233		No virus	No drug
100 µM	1.176	1.189	2.236	0.542
25 µM	1.009	0.889	2.107	0.588
	No virus	No drug	No virus	No drug
	2.313	0.527	2.405	0.577
	2.353	0.532	2.371	0.681

B

	2'-fluoro-2'-deoxycytidine			
Inhibitor (nM)	Absorbance @ 450 nm			Control wells
10	0.915	1.192	0.955	Virus no drug
26	1.177	0.926	0.867	1.322
66	0.958	1.224	0.915	1.19
164	1.057	0.861	0.934	1.019
410	0.981	1.372	1.15	0.959
1024	1.167	1.134	1.048	No virus no drug
2560	1.535	1.824	1.994	2.701
6400	2.424	2.198	2.035	2.646
16000	2.658	2.484	2.59	2.688
40000	2.624	2.53	2.606	2.738
100000	2.706	2.55	2.549	

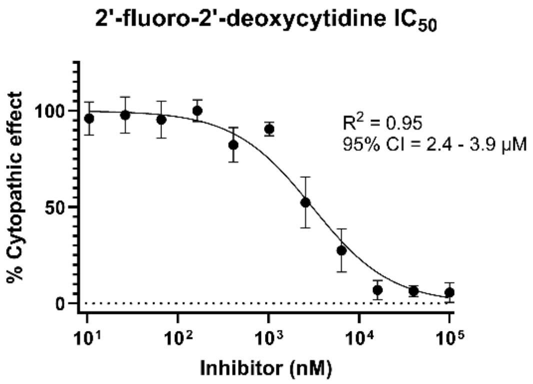

	N4-benzoyl-2'-fluoro-2'-deoxycytidine			
Inhibitor (nM)	Absorbance @ 450 nm			
98	0.862	0.933	0.728	Control wells
195	0.854	0.777	0.892	Virus no drug
391	0.812	0.819	0.907	0.799
781	0.819	0.974	0.78	0.769
1563	1.002	0.945	0.999	0.652
3125	0.784	0.868	0.908	0.699
6250	0.938	1.122	0.969	No virus no drug
12500	1.129	1.092	1.003	2.65
25000	1.153	1.121	1.273	2.497
50000	2.114	2.105	2.15	2.469
100000	2.317	2.333	2.319	2.445
200000	2.178	2.19	2.166	

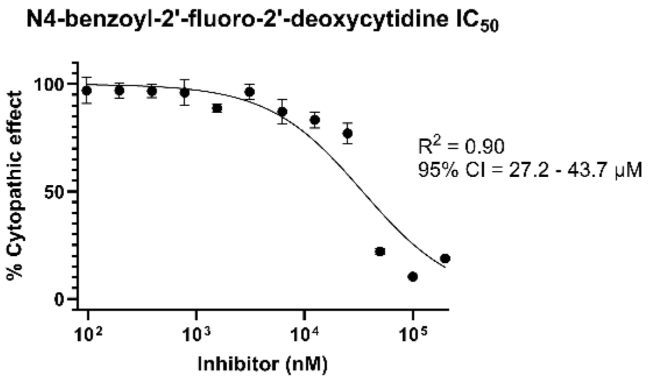

	N4-acetyl-2'-fluoro-2'-deoxycytidine			
Inhibitor (nM)	Absorbance @ 450 nm			
98	0.869	0.677	0.66	Control wells
195	0.814	0.877	0.958	Virus no drug
391	0.741	0.75	0.86	0.799
781	0.925	0.881	0.781	0.769
1563	1.017	0.954	1.071	0.652
3125	0.994	0.896	0.717	0.699
6250	0.892	0.954	1.11	No virus no drug
12500	1.202	1.117	1.036	2.65
25000	2.041	1.813	1.84	2.497
50000	2.416	2.336	2.254	2.469
100000	2.503	2.29	2.184	2.445
200000	2.135	2.086	2.06	

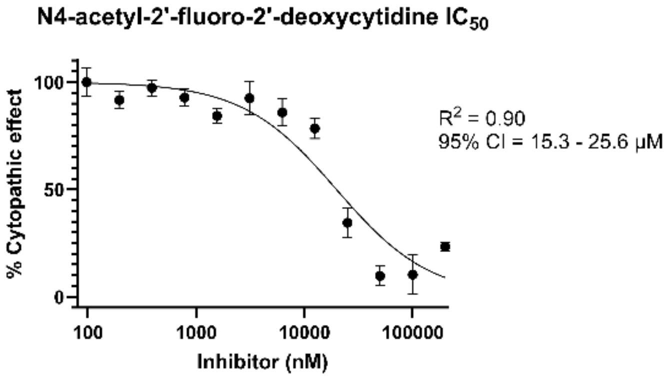

	Favipiravir			
Inhibitor (nM)	Absorbance @ 450 nm			
98	0.741	0.706	0.889	Control wells
195	0.776	0.969	0.769	Virus no drug
391	0.639	0.875	1.033	0.73
781	0.755	1.133	0.729	0.785
1563	0.93	1.042	1.129	0.71
3125	0.679	0.794	1.02	0.737
6250	0.866	0.939	0.995	No virus no drug
12500	1.153	0.991	1.047	2.592
25000	0.939	1.093	1.158	2.59
50000	1.539	1.878	1.268	2.584
100000	2.573	2.378	2.42	2.584
200000	2.395	2.191	2.263	

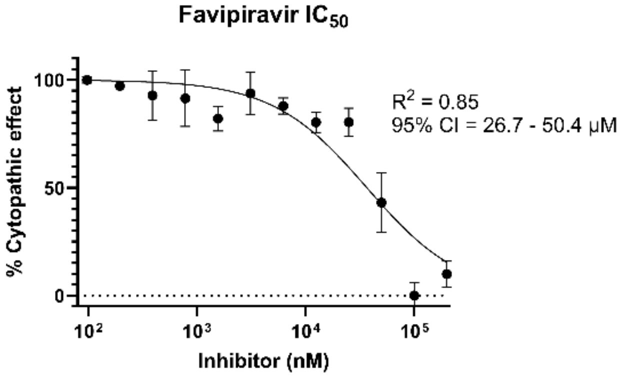

	EIDD-1931			
Inhibitor (nM)	Absorbance @ 450 nm			Control wells
4.2	0.859	1.001	1.053	Virus no drug
10.5	1.098	1.079	1.035	1.002
26.2	0.91	1.128	1.134	0.883
65.5	0.875	0.895	1.035	1.018
163.8	0.874	1.124	1.194	0.915
409.6	1.197	1.134	0.791	No virus no drug
1024.0	1.505	1.048	1.153	2.691
2560.0	2.126	1.984	2.155	2.701
6400.0	2.622	2.613	2.634	2.682
16000.0	2.641	2.51	2.559	2.712
40000.0	2.451	2.459	2.421	
100000.0	2.532	2.574	2.564	

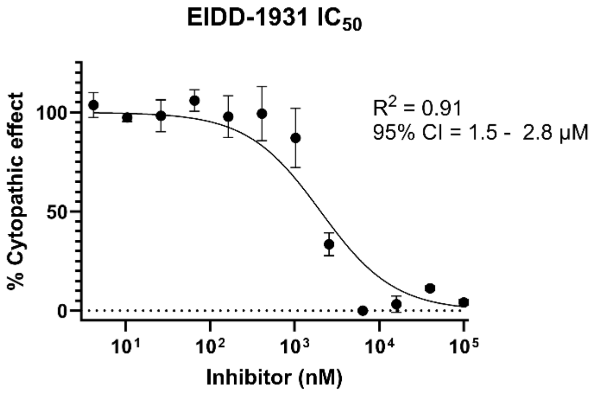

	Molnupiravir			
Inhibitor (nM)	Absorbance @ 450 nm			
98	0.745	0.852	0.841	Control wells
195	0.902	0.923	0.735	Virus no drug
391	0.665	0.815	0.881	0.676
781	0.653	1.093	0.835	0.767
1563	0.711	0.964	0.934	0.712
3125	0.722	0.896	0.9	0.76
6250	0.723	0.922	0.779	No virus no drug
12500	1.147	0.875	1.204	2.476
25000	1.45	1.294	1.483	2.52
50000	2.555	2.362	2.285	2.551
100000	2.603	2.396	2.312	2.55
200000	2.457	2.296	2.339	

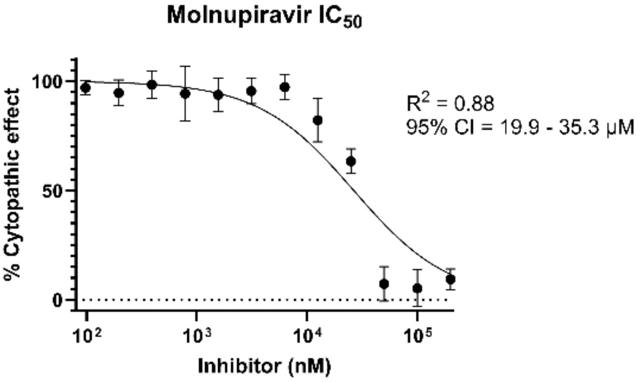

	Ribavirin			
Inhibitor (nM)	Absorbance @ 450 nm			
98	0.93	0.808	0.846	Control wells
195	1.028	0.751	1.077	Virus no drug
391	1.079	1.106	0.975	0.624
781	0.831	0.772	0.942	0.652
1563	0.824	0.983	0.887	0.77
3125	0.811	0.751	0.919	0.699
6250	1.473	0.903	0.885	No virus no drug
12500	0.944	1.019	0.964	2.506
25000	1.252	1.069	1.079	2.501
50000	1.485	1.539	2.098	2.51
100000	2.428	1.77	2.235	2.535
200000	1.6	1.923	1.881	

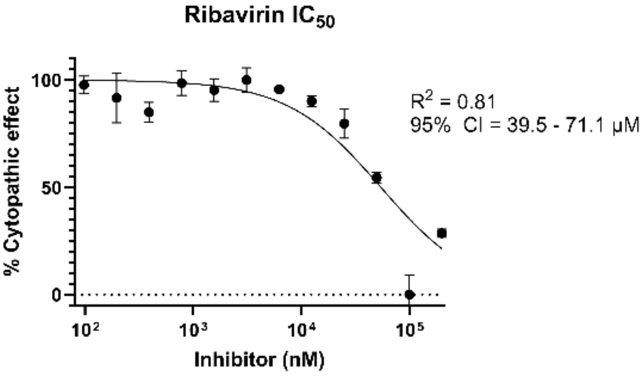

	2'-fluoro-2'-deoxyguanine			
Inhibitor (nM)	Absorbance @ 450 nm			
98	0.93	1.294	0.816	Control wells
195	0.879	0.953	0.981	Virus no drug
391	0.962	1.245	0.935	0.624
781	0.973	0.946	0.836	0.652
1563	0.829	1	1.026	0.77
3125	0.953	0.929	0.996	0.699
6250	1.09	1.022	1.12	No virus no drug
12500	1.319	1.108	1.111	2.506
25000	1.365	1.382	1.545	2.501
50000	1.857	1.786	1.984	2.51
100000	2.352	2.27	2.333	2.535
200000	1.993	2.01	2.333	

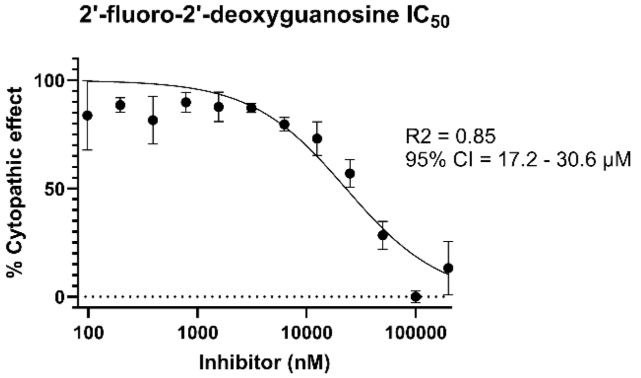

Figure S1. (**A**) Raw datasets and bar graphs from the primary screen of the inhibitor set. Compounds were tested at 25 µM (blue) and 100 µM (green). Inhibitors highlighted in red reduced cytopathic effect (CPE) to <50% at 100 µM and were selected for dose–response titration to determine IC_{50} values. The primary screen was performed in biological duplicates. (**B**) Raw datasets and titration curves for the set of inhibitors that have shown activity in the cytopathic effect reduction assay. All experiments were performed in biological triplicates and titration curves were fitted using nonlinear regression as inhibitor concentration versus normalized response, with 95% confidence intervals calculated.

	EIDD-1931			Uninhibited
Inhibitor (nM)	Firefly luciferase/β-Galactosidase ratio			polymerase
100000	38.83495	39.52569	172.3519	21522.27
40000	41.32231	192.8721	226.4151	13488
16000	677.4194	977.5967	1211.215	19908.05
6400	3237.209	5090.909	5344.538	Full inhibition
2560	7840	6557.522	10447.15	
1024	12780.68	10688.8	13101.34	38.83495
410	12463.07	23116.88	14060.15	39.52569
164	21522.27	13488	19908.05	172.3519

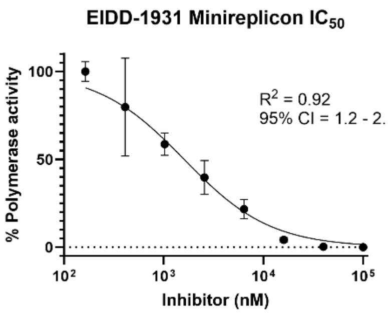

	2'-fluoro-2'-deoxycytidine			Uninhibited
Inhibitor (nM)	Firefly luciferase/β-Galactosidase ratio			polymerase
100000	70.17544	198.4733	76.92308	15201.65
40000	651.7312	1077.228	112.2244	14650.51
16000	1432.099	3632	94.86166	13984.06
6400	8208.617	6635.514	8433.498	Full inhibition
2560	13621.15	9333.333	14916.84	
1024	20580.65	12585.57	16179.96	70.17544
410	13949.58	17187.88	16485.09	198.4733
164	15201.65	14650.51	13984.06	76.92308

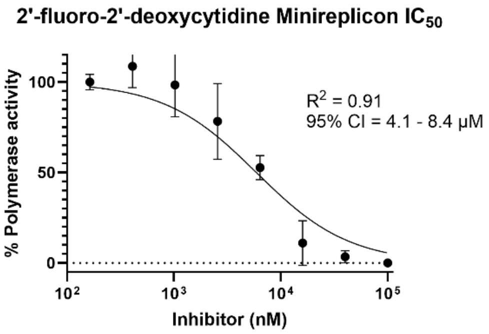

Figure S2. Raw datasets and titration curves for the pair of inhibitors evaluated by the minireplicon assay. Numbers in both datasets indicate ratio of relative luminescence units (RLU) and absorbance at 450 nm as detailed in Experimental section. All experiments were performed in biological triplicates, titration curves were fitted using nonlinear regression as inhibitor concentration versus normalized response, with 95% confidence intervals calculated. As negative control, the plasmid encoding the RVFV L protein was omitted from the transfection.

	2'-fluoro-2'-deoxycytidine				
Inhibitor (nM)	Absorbance @ 450 nm			DMSO ctrl	No drug ctrl
97.7	2.669	2.531	2.682	0.289	2.449
195.3	2.567	2.513	2.585	0.295	2.836
390.6	2.533	2.394	2.627	0.29	2.761
781.3	2.507	2.403	2.573	0.289	2.689
1562.5	2.457	2.42	2.597	0.291	2.734
3125.0	2.432	2.425	2.562	0.293	2.763
6250.0	2.469	2.397	2.556	0.287	2.753
12500.0	2.502	2.43	2.558	0.291	2.589
25000.0	2.363	2.41	2.497	0.292	2.713
50000.0	2.49	2.469	2.6	0.293	2.44
100000.0	2.058	2.139	2.31	0.3	2.737
200000.0	2.195	2.225	2.318	0.311	2.678

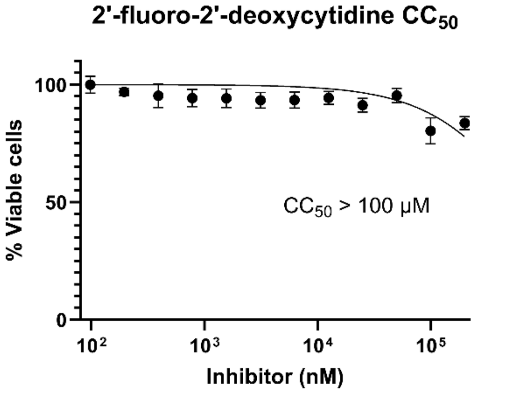

	EIDD-1931				
Inhibitor (nM)	Absorbance @ 450 nm			DMSO ctrl	No drug ctrl
97.7	2.739	2.735	2.728	0.289	2.449
195.3	2.625	2.557	2.554	0.295	2.836
390.6	2.59	2.558	2.569	0.29	2.761
781.3	2.627	2.605	2.564	0.289	2.689
1562.5	2.558	2.612	2.468	0.291	2.734
3125.0	2.585	2.52	2.514	0.293	2.763
6250.0	2.596	2.589	2.445	0.287	2.753
12500.0	2.569	2.484	2.533	0.291	2.589
25000.0	2.539	2.514	2.527	0.292	2.713
50000.0	2.497	2.489	2.417	0.293	2.44
100000.0	2.405	2.349	2.343	0.3	2.737
200000.0	2.038	2.153	2.016	0.311	2.678

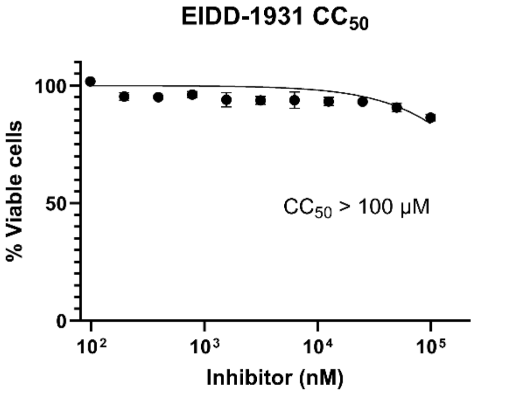

	Ribavirin				
Inhibitor (nM)	Absorbance @ 450 nm			DMSO ctrl	No drug ctrl
97.7	2.739	2.735	2.728	0.288	2.494
195.3	2.625	2.557	2.554	0.286	2.131
390.6	2.59	2.558	2.569	0.29	2.051
781.3	2.627	2.605	2.564	0.291	2.146
1562.5	2.558	2.612	2.468	0.288	2.251
3125.0	2.585	2.52	2.514	0.286	2.105
6250.0	2.596	2.589	2.445	0.289	2.432
12500.0	2.569	2.484	2.533	0.291	2.395
25000.0	2.539	2.514	2.527	0.29	2.351
50000.0	2.497	2.489	2.417	0.292	2.344
100000.0	2.405	2.349	2.343	0.295	2.391
200000.0	2.038	2.153	2.016	0.303	2.514

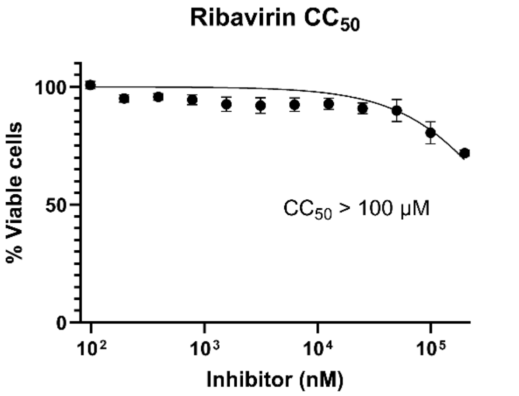

	N4-acetyl-2'-fluoro-2'-deoxycytidine				
Inhibitor (nM)	Absorbance @ 450 nm			DMSO ctrl	No drug ctrl
97.7	2.743	2.721	2.755	0.288	2.494
195.3	2.628	2.586	2.604	0.286	2.131
390.6	2.634	2.578	2.536	0.29	2.051
781.3	2.612	2.554	2.536	0.291	2.146
1562.5	2.603	2.534	2.513	0.288	2.251
3125.0	2.641	2.547	2.521	0.286	2.105
6250.0	2.635	2.614	2.506	0.289	2.432
12500.0	2.606	2.508	2.521	0.291	2.395
25000.0	2.602	2.454	2.385	0.29	2.351
50000.0	2.453	2.504	2.395	0.292	2.344
100000.0	2.286	2.291	2.327	0.295	2.391
200000.0	2.001	1.913	2.019	0.303	2.514

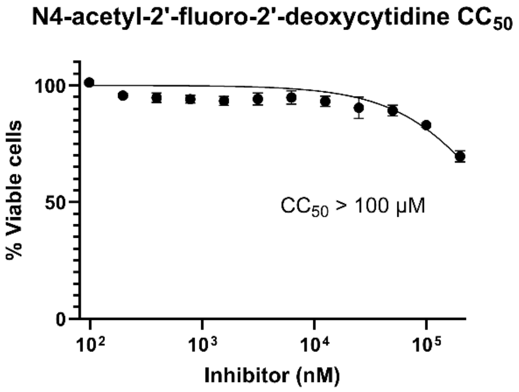

	N4-benzoyl-2'-fluoro-2'-deoxycytidine				
Inhibitor (nM)	Absorbance @ 450 nm			DMSO ctrl	No drug ctrl
97.7	2.699	2.661	2.678	0.278	2.958
195.3	2.587	2.491	2.595	0.302	2.832
390.6	2.571	2.547	2.56	0.281	2.661
781.3	2.542	2.538	2.591	0.286	2.892
1562.5	2.547	2.484	2.536	0.292	2.866
3125.0	2.496	2.565	2.582	0.286	2.565
6250.0	2.525	2.517	2.62	0.291	2.827
12500.0	2.452	2.515	2.559	0.284	2.602
25000.0	2.533	2.465	2.522	0.279	2.527
50000.0	2.463	2.45	2.48	0.299	2.643
100000.0	2.283	2.199	2.343	0.296	2.804
200000.0	1.987	1.934	1.825	0.316	2.886

N4-benzoyl-2'-fluoro-2'-deoxycytidine CC_{50}

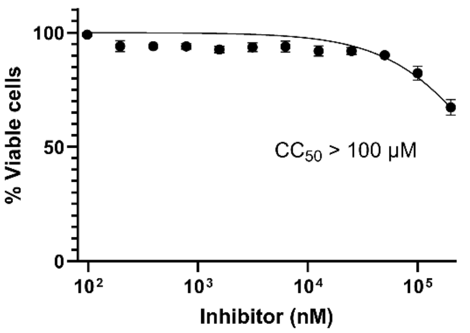

	Favipiravir				
Inhibitor (nM)	Absorbance @ 450 nm			DMSO ctrl	No drug ctrl
97.7	2.744	2.74	2.638	0.278	2.958
195.3	2.682	2.62	2.566	0.302	2.832
390.6	2.64	2.607	2.531	0.281	2.661
781.3	2.636	2.534	2.469	0.286	2.892
1562.5	2.604	2.54	2.481	0.292	2.866
3125.0	2.638	2.534	2.419	0.286	2.565
6250.0	2.626	2.606	2.464	0.291	2.827
12500.0	2.586	2.512	2.456	0.284	2.602
25000.0	2.475	2.481	2.409	0.279	2.527
50000.0	2.405	2.36	2.393	0.299	2.643
100000.0	2.197	2.209	2.146	0.296	2.804
200000.0	1.777	1.818	1.944	0.316	2.886

Favipiravir CC_{50}

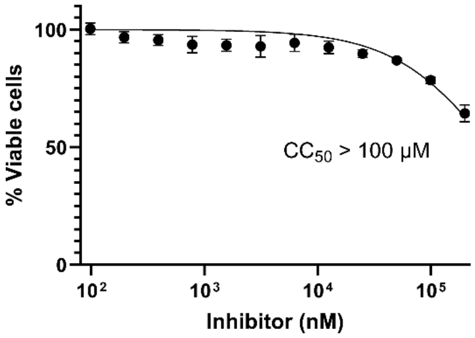

	Molnupiravir				
Inhibitor (nM)	Absorbance @ 450 nm			DMSO ctrl	No drug ctrl
97.7	2.766	2.702	2.749	0.295	2.904
195.3	2.617	2.592	2.634	0.306	2.819
390.6	2.616	2.589	2.647	0.305	2.893
781.3	2.541	2.564	2.64	0.302	2.731
1562.5	2.558	2.573	2.598	0.299	2.515
3125.0	2.448	2.639	2.607	0.298	2.408
6250.0	2.434	2.579	2.605	0.295	2.68
12500.0	2.411	2.51	2.592	0.297	2.489
25000.0	2.433	2.473	2.535	0.298	2.595
50000.0	2.367	2.419	2.498	0.313	2.583
100000.0	2.202	2.207	2.345	0.304	2.229
200000.0	2.133	2.295	2.269	0.306	2.632

Molnupiravir CC_{50}

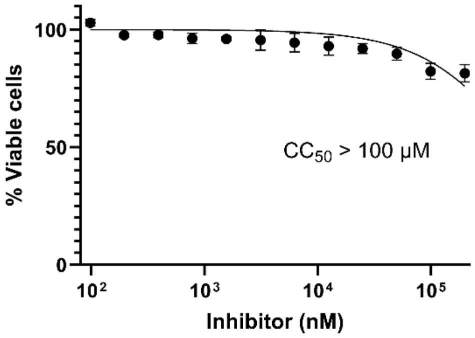

	2'-fluoro-2'-deoxyguanosine				
Inhibitor (nM)	Absorbance @ 450 nm			DMSO ctrl	No drug ctrl
97.7	2.739	2.735	2.728	0.295	2.904
195.3	2.625	2.557	2.554	0.306	2.819
390.6	2.59	2.558	2.569	0.305	2.893
781.3	2.627	2.605	2.564	0.302	2.731
1562.5	2.558	2.612	2.468	0.299	2.515
3125.0	2.585	2.52	2.514	0.298	2.408
6250.0	2.596	2.589	2.445	0.295	2.68
12500.0	2.569	2.484	2.533	0.297	2.489
25000.0	2.539	2.514	2.527	0.298	2.595
50000.0	2.497	2.489	2.417	0.313	2.583
100000.0	2.405	2.349	2.343	0.304	2.229
200000.0	2.038	2.153	2.016	0.306	2.632

2'-fluoro-2'-deoxyguanosine CC_{50}

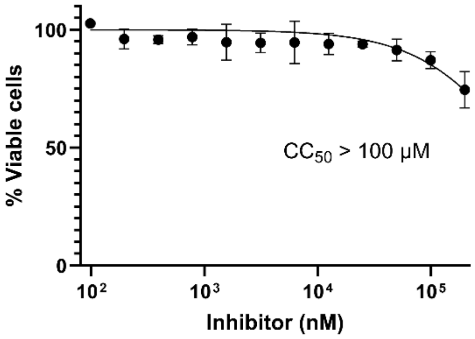

	T-1105				
Inhibitor (nM)	Absorbance @ 450 nm			DMSO control	No drug control
100000.0	2.205	2.25	2.165	0.674	2.457
40000.0	2.318	1.949	2.053	1.042	2.373
16000.0	2.132	2.099	1.971	0.751	2.358
6400.0	2.137	2.265	2.2	1.123	2.514
2560.0	2.196	2.128	2.24	0.639	2.378
1024.0	2.2	2.253	2.219	0.923	2.44
409.6	2.187	2.355	2.297	0.653	2.458
163.8	2.406	2.436	2.341	0.664	2.565

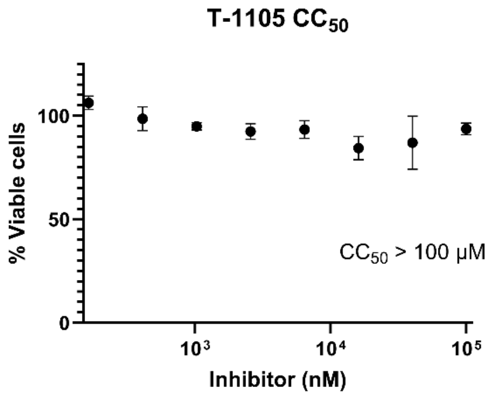

	2'-fluoro-2'-deoxyadenine				
Inhibitor (nM)	Absorbance @ 450 nm			DMSO ctrl	No drug ctrl
100000.0	2.497	2.196	2.204	0.751	2.462
40000.0	2.418	2.166	2.181	0.7	2.441
16000.0	2.43	2.169	2.051	0.76	2.135
6400.0	2.423	2.013	2.053	0.735	2.309
2560.0	2.374	2.317	2.222	0.573	2.405
1024.0	2.154	2.209	2.122	0.699	2.196
409.6	2.352	2.229	2.122	0.739	2.287
163.8	2.433	2.248	2.119	0.629	2.306

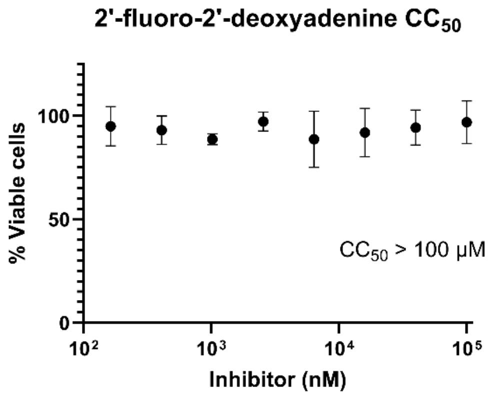

	2'-fluoro-2'-deoxythymidine				
Inhibitor (nM)	Absorbance @ 450 nm			DMSO ctrl	No drug ctrl
100000.0	2.211	2.126	2.19	0.751	2.462
40000.0	2.197	1.849	1.999	0.7	2.441
16000.0	2.202	2.099	1.952	0.76	2.135
6400.0	2.148	2.143	2.013	0.735	2.309
2560.0	2.111	2.27	2.258	0.573	2.405
1024.0	2.016	2.083	2.168	0.699	2.196
409.6	2.222	2.144	2.257	0.739	2.287
163.8	2.38	2.211	2.294	0.629	2.306

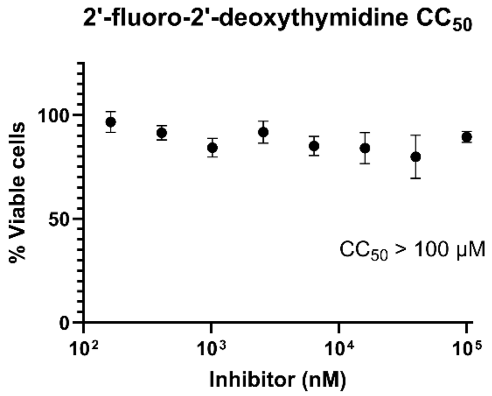

	Dasabuvir				
Inhibitor (nM)	Absorbance @ 450 nm			DMSO ctrl	No drug ctrl
100000.0	0.39	0.378	0.367	1.133	2.492
40000.0	0.351	0.38	0.406	1.282	2.375
16000.0	2.349	2.374	2.242	0.938	2.232
6400.0	2.297	2.205	2.128	0.951	2.212
2560.0	2.351	2.335	2.108	0.821	2.238
1024.0	2.27	2.138	2.017	0.983	2.009
409.6	2.383	2.164	2.155	1.034	2.148
163.8	2.292	2.275	2.263	1.056	2.257

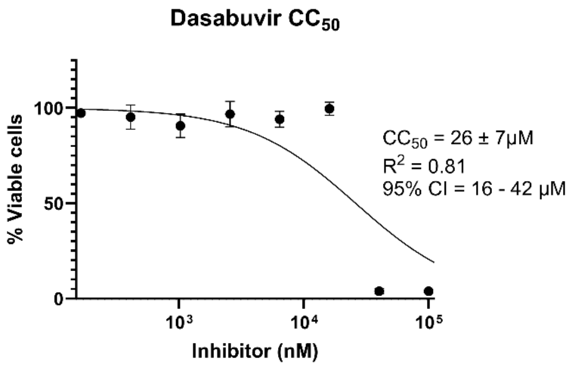

	Lamivudine				
Inhibitor (nM)	Absorbance @ 450 nm			DMSO ctrl	No drug ctrl
100000.0	2.059	2.022	2.062	0.751	2.462
40000.0	2.45	2.456	2.421	0.7	2.441
16000.0	2.085	2.086	2.133	0.76	2.135
6400.0	2.317	2.392	2.473	0.735	2.309
2560.0	2.256	2.398	2.395	0.573	2.405
1024.0	2.383	2.322	2.266	0.699	2.196
409.6	2.315	2.327	2.394	0.739	2.287
163.8	2.343	2.403	2.233	0.629	2.306

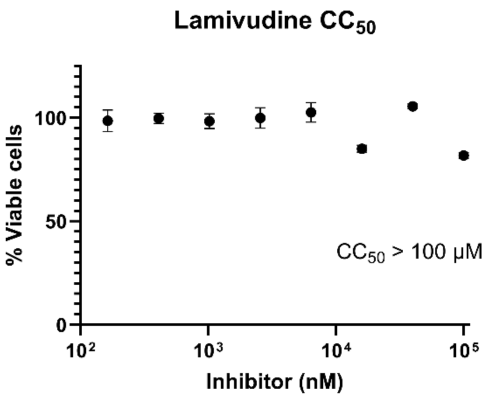

	PSI-7977 (Sofosbuvir)				
Inhibitor (nM)	Absorbance @ 450 nm			DMSO ctrl	No drug ctrl
100000.0	2.133	2.454	2.223	1.133	2.719
40000.0	2.276	2.216	2.29	1.282	2.414
16000.0	2.107	2.225	2.246	0.938	2.275
6400.0	2.169	2.263	2.162	0.951	2.355
2560.0	2.083	2.188	2.129	0.821	2.309
1024.0	1.987	2.018	2.035	0.983	2.45
409.6	2.019	2.102	2.141	1.034	2.35
163.8	2.212	2.229	2.237	1.056	2.396

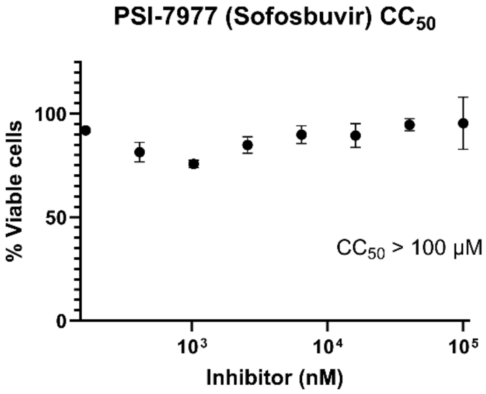

	Baloxavir Marboxil				
Inhibitor (nM)	Absorbance @ 450 nm			DMSO ctrl	No drug ctrl
100000.0	2.102	1.753	2.102	0.722	2.185
40000.0	1.766	1.875	2.07	0.838	2.215
16000.0	2.458	2.196	2.172	0.822	2.354
6400.0	2.129	2.082	2.108	0.798	2.097
2560.0	2.349	2.109	2.126	0.989	2.249
1024.0	2.174	2.236	2.146	0.736	1.891
409.6	2.26	2.227	2.136	0.716	1.956
163.8	2.251	2.267	2.223	0.614	2.054

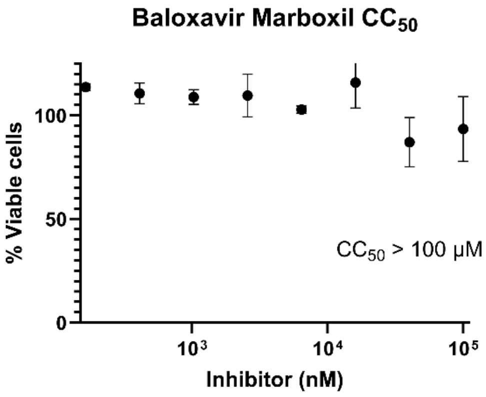

	VX-787				
Inhibitor (nM)	Absorbance @ 450 nm			DMSO ctrl	No drug ctrl
100000.0	1.923	1.974	1.994	0.722	2.185
40000.0	2.16	1.986	2.017	0.838	2.215
16000.0	2.072	2.17	2.113	0.822	2.354
6400.0	2.077	2.201	2.05	0.798	2.097
2560.0	2.11	2.046	2.135	0.989	2.249
1024.0	2.055	2.061	2.038	0.736	1.891
409.6	2.127	2.129	2.134	0.716	1.956
163.8	2.215	2.14	2.054	0.614	2.054

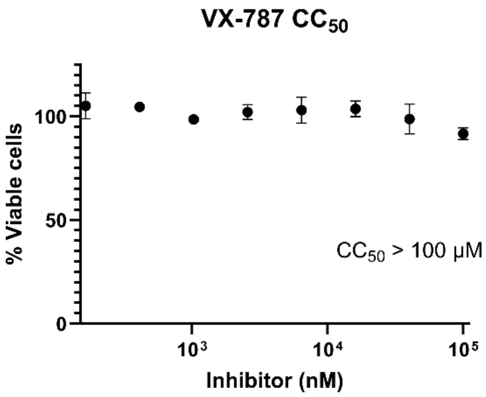

	Cytosine arabinoside				
Inhibitor (nM)	Absorbance @ 450 nm			DMSO ctrl	No drug ctrl
200000	1.673	1.646	1.712	0.27	2.658
100000	2.185	2.109	2.193	0.261	2.555
50000	2.246	1.927	2.187	0.266	2.536
25000	2.277	2.207	2.284	0.265	2.47
12500	2.423	2.322	2.237	0.266	2.393
6250	2.362	2.205	2.184	0.264	2.435
3125	2.484	2.42	2.394	0.266	2.554
1562.5	2.522	2.447	2.471	0.278	2.644
781.3	2.51	2.551	2.542		
390.6	2.588	2.459	2.508		
195.3	2.545	2.599	2.48		
97.7	2.558	2.644	2.495		

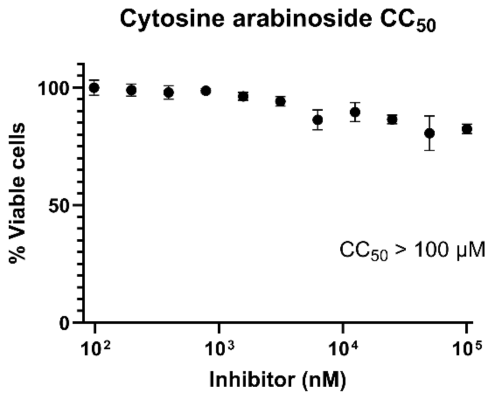

	Remdesivir				
Inhibitor (nM)	Absorbance @ 450 nm			DMSO ctrl	No drug ctrl
100000.0	1.45	1.438	1.302	0.647	2.252
40000.0	1.447	1.39	1.65	0.632	2.079
16000.0	1.689	1.695	1.79	0.619	1.794
6400.0	1.606	1.759	1.731	0.566	1.957
2560.0	1.819	1.899	1.892	0.515	1.756
1024.0	1.799	1.814	1.813	0.463	1.968
409.6	1.844	1.928	1.865	0.759	1.83
163.8	1.821	1.83	1.764	0.555	1.632

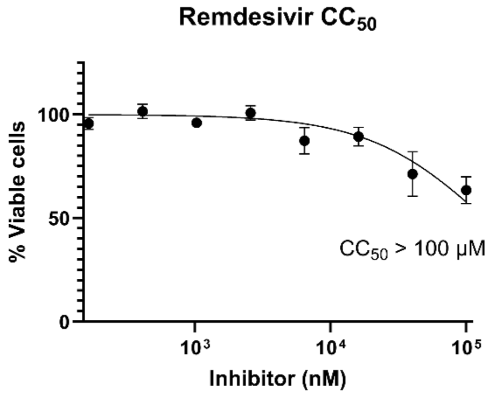

	LY-2334737				
Inhibitor (nM)	Absorbance @ 450 nm			DMSO ctrl	No drug ctrl
200000.0	1.628	1.663	1.452	0.276	2.875
80000.0	2.317	2.252	2.369	0.253	2.767
32000.0	2.365	2.394	2.21	0.276	2.784
12800.0	2.357	2.277	2.232	0.257	2.738
5120.0	2.436	2.448	2.472	0.263	2.729
2048.0	2.513	2.661	2.512	0.257	2.748
819.2	2.69	2.66	2.599	0.283	2.718
327.7	2.793	2.862	2.771	0.255	2.837

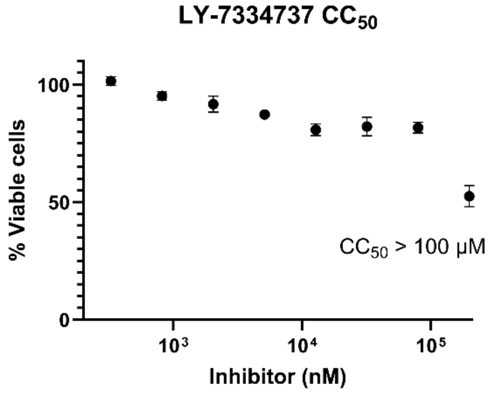

	Gemcitabine Elaidate				
Inhibitor (nM)	Absorbance @ 450 nm			DMSO ctrl	No drug ctrl
100000.0	2.82	2.822	2.886	0.751	2.824
40000.0	2.7	2.696	2.842	0.7	2.717
16000.0	2.751	2.742	2.815	0.76	2.791
6400.0	2.706	2.71	2.846	0.735	2.695
2560.0	2.731	2.731	2.806	0.573	2.747
1024.0	2.714	2.718	2.846	0.699	2.74
409.6	2.705	2.707	2.795	0.739	2.692
163.8	2.834	2.794	2.914	0.629	2.724

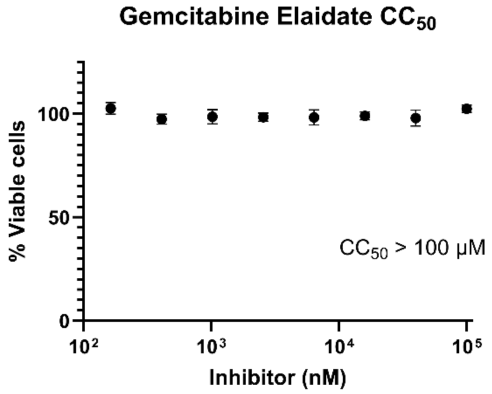

	PSI-6130				
Inhibitor (nM)	Absorbance @ 450 nm			DMSO ctrl	No drug ctrl
100000.0	2.027	1.926	1.907	0.53	2.206
40000.0	2.062	2.091	2.053	0.481	2.096
16000.0	2.131	2.053	1.911	0.48	1.933
6400.0	2.108	2.011	1.886	0.466	1.947
2560.0	2.18	2.055	2.114	0.481	1.94
1024.0	1.997	2.007	2.017	0.555	1.957
409.6	2.022	2.033	1.984	0.513	1.964
163.8	1.796	2.113	2.102	0.556	2.194

PSI-6130 CC_{50}

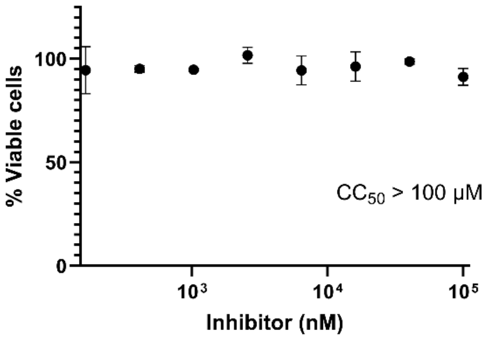

	Cyclopentenyluracil				
Inhibitor (nM)	Absorbance @ 450 nm			DMSO ctrl	No drug ctrl
100000.0	1.929	1.981	1.961	0.53	2.206
40000.0	2.001	1.993	1.997	0.481	2.096
16000.0	1.928	1.801	1.817	0.48	1.933
6400.0	1.986	1.958	1.85	0.466	1.947
2560.0	1.937	1.958	1.971	0.481	1.94
1024.0	1.955	1.9	1.841	0.555	1.957
409.6	1.982	1.736	1.949	0.513	1.964
163.8	2.098	1.95	2.177	0.556	2.194

Cyclopentenyluracil CC_{50}

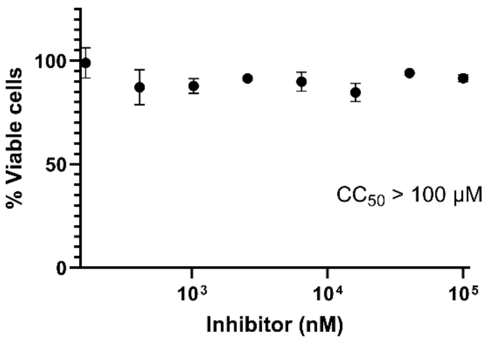

	Cidofovir				
Inhibitor (nM)	Absorbance @ 450 nm			DMSO ctrl	No drug ctrl
100000.0	2.368	2.374	2.282	0.7	2.322
40000.0	2.397	2.361	2.186	0.654	2.335
16000.0	2.305	2.277	2.042	0.821	2.245
6400.0	2.21	2.232	2.144	0.646	2.016
2560.0	2.249	2.001	2.024	0.636	2.158
1024.0	2.18	2.121	2.135	0.593	2.025
409.6	2.129	2.176	1.899	0.603	1.976
163.8	2.207	2.123	2.198	0.586	2.112

Cidofovir CC_{50}

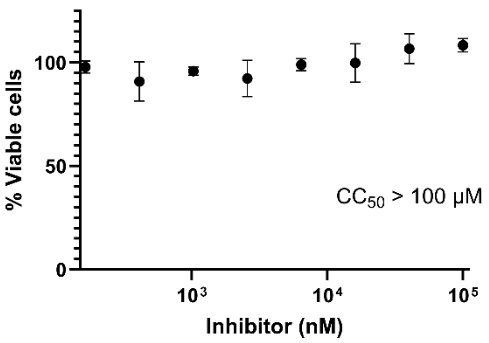

	Tenofovir fumarate				
Inhibitor (nM)	Absorbance @ 450 nm			DMSO ctrl	No drug ctrl
100000.0	2.296	2.227	2.247	0.7	2.322
40000.0	2.322	2.103	2.315	0.654	2.335
16000.0	2.067	2.153	1.818	0.821	2.245
6400.0	1.947	2.166	2.163	0.646	2.016
2560.0	2.115	2.009	1.918	0.636	2.158
1024.0	2.014	2.164	1.838	0.593	2.025
409.6	2.034	1.988	2.01	0.603	1.976
163.8	2.106	2.101	2.141	0.586	2.112

Tenofovir fumarate CC_{50}

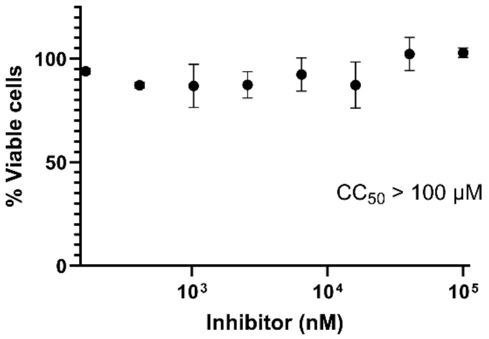

	Gemcitabine				
Inhibitor (nM)	Absorbance @ 450 nm			DMSO ctrl	No drug ctrl
100000.0	2.797	2.805	2.789	0.751	2.824
40000.0	2.71	2.688	2.744	0.7	2.717
16000.0	2.841	2.73	2.744	0.76	2.791
6400.0	2.68	2.68	2.714	0.735	2.695
2560.0	2.723	2.744	2.741	0.573	2.747
1024.0	2.702	2.696	0.547	0.699	2.74
409.6	2.73	2.737	2.738	0.739	2.692
163.8	2.801	2.794	2.804	0.629	2.724

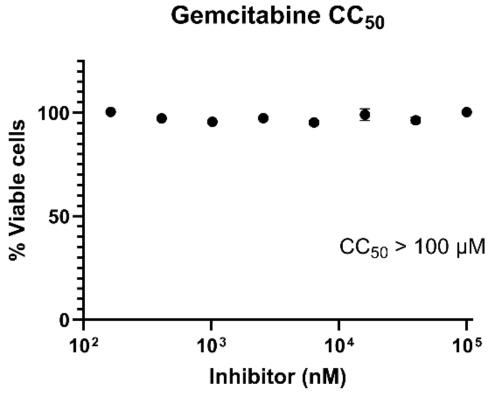

	Stavudine (d4T)				
Inhibitor (nM)	Absorbance @ 450 nm			DMSO ctrl	No drug ctrl
100000	2.461	2.31	2.179	0.413	2.573
40000	2.516	2.241	2.098	0.389	2.608
16000	2.392	2.221	1.982	0.519	2.401
6400	2.394	2.134	2.292	0.5	2.361
2560	2.449	2.345	2.237	0.46	2.406
1024	2.39	2.357	2.116	0.484	2.393
409.6	2.425	2.308	2.303	0.49	2.451
163.8	2.089	2.432	2.46	0.47	2.536

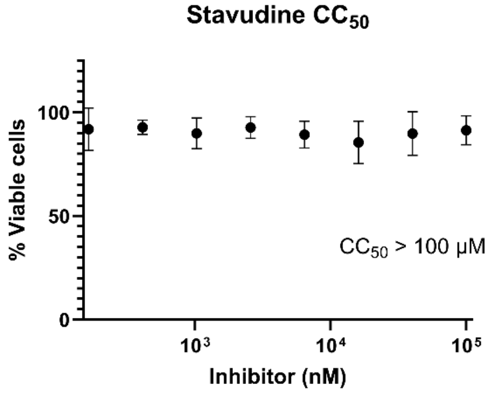

	Emtricitabine (FTC)				
Inhibitor (nM)	Absorbance @ 450 nm			DMSO ctrl	No drug ctrl
100000	1.986	2.166	2.07	0.413	2.573
40000	2.236	2.306	2.274	0.389	2.608
16000	2.048	2.123	2.171	0.519	2.401
6400	2.038	2.186	2.16	0.5	2.361
2560	2.34	2.231	2.215	0.46	2.406
1024	2.273	2.165	2.084	0.484	2.393
409.6	2.291	2.313	2.217	0.49	2.451
163.8	2.716	2.431	2.45	0.47	2.536

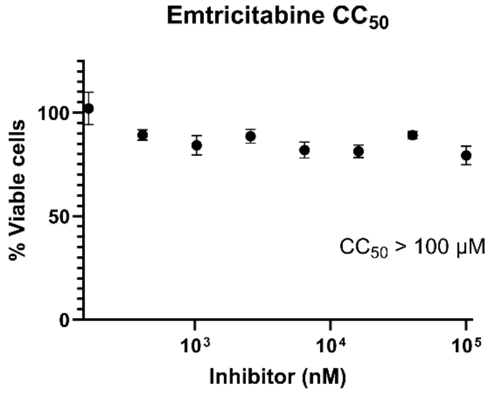

	Zidovudine (AZT)				
Inhibitor (nM)	Absorbance @ 450 nm			DMSO ctrl	No drug ctrl
100000	2.064	2.197	2.278	0.413	2.573
40000	2.33	2.366	2.302	0.389	2.608
16000	2.118	2.129	2.236	0.519	2.401
6400	2.1	2.123	2.203	0.5	2.361
2560	2.25	2.265	2.213	0.46	2.406
1024	2.215	2.274	2.239	0.484	2.393
409.6	2.204	2.297	2.355	0.49	2.451
163.8	2.513	2.463	2.394	0.47	2.536

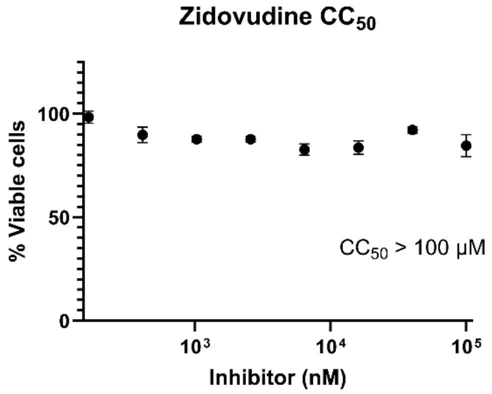

	Cyclopentenylcytosine				
Inhibitor (nM)	Absorbance @ 450 nm			DMSO ctrl	No drug ctrl
100000	1.979	1.807	1.838	0.26	2.573
40000	2.305	2.416	1.913	0.209	2.608
16000	2.343	1.981	1.962	0.301	2.401
6400	2.252	1.962	2.007	0.243	2.361
2560	2.244	2.067	1.979	0.264	2.406
1024	2.166	1.991	1.939	0.242	2.393
409.6	2.168	2.019	2.173	0.271	2.451
163.8	1.971	2.125	2.245	0.236	2.536

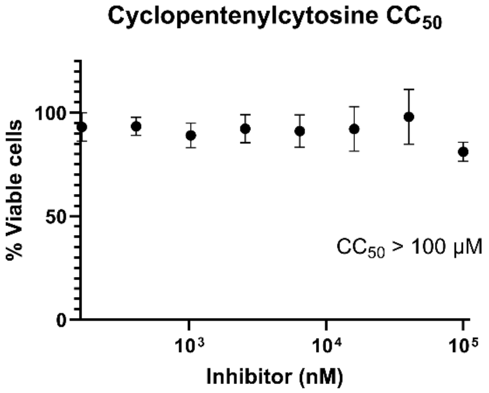

	5'-azacytidine				
Inhibitor (nM)	Absorbance @ 450 nm			DMSO ctrl	No drug ctrl
100000.0	1.938	1.972	1.948	0.592	2.342
40000.0	1.952	1.908	1.901	0.673	2.387
16000.0	1.864	1.907	1.962	0.689	2.49
6400.0	1.889	2.079	2.005	0.617	2.581
2560.0	1.972	1.855	1.917	0.641	2.476
1024.0	1.957	2.038	1.988	0.671	2.419
409.6	2.139	2.124	2.233	0.663	2.553
163.8	2.116	2.143	2.118	0.654	2.512

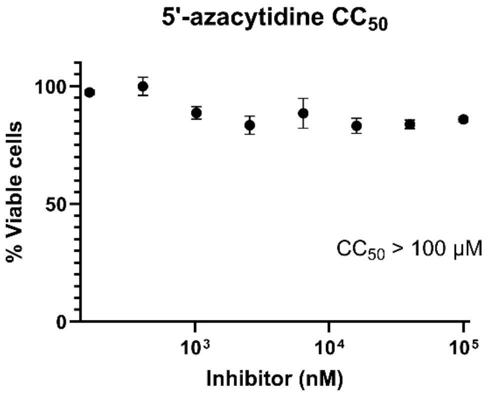

	5'-fluoro-2'-deoxycytidine				
Inhibitor (nM)	Absorbance @ 450 nm			DMSO ctrl	No drug ctrl
200000.0	1.628	1.663	1.452	0.276	2.875
80000.0	2.317	2.252	2.369	0.253	2.767
32000.0	2.365	2.394	2.21	0.276	2.784
12800.0	2.357	2.277	2.232	0.257	2.738
5120.0	2.436	2.448	2.472	0.263	2.729
2048.0	2.513	2.661	2.512	0.257	2.748
819.2	2.69	2.66	2.599	0.283	2.718
327.7	2.793	2.862	2.771	0.255	2.837

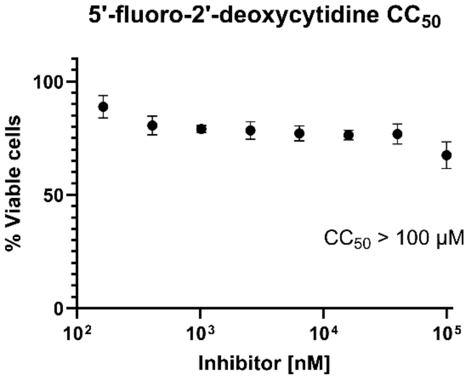

Figure S3. Cytotoxicity evaluation and titration curves of the inhibitors identified during the initial screening. All experiments were performed in biological triplicates, titration curves were fitted using nonlinear regression as inhibitor concentration versus normalized response, with 95% confidence intervals calculated.

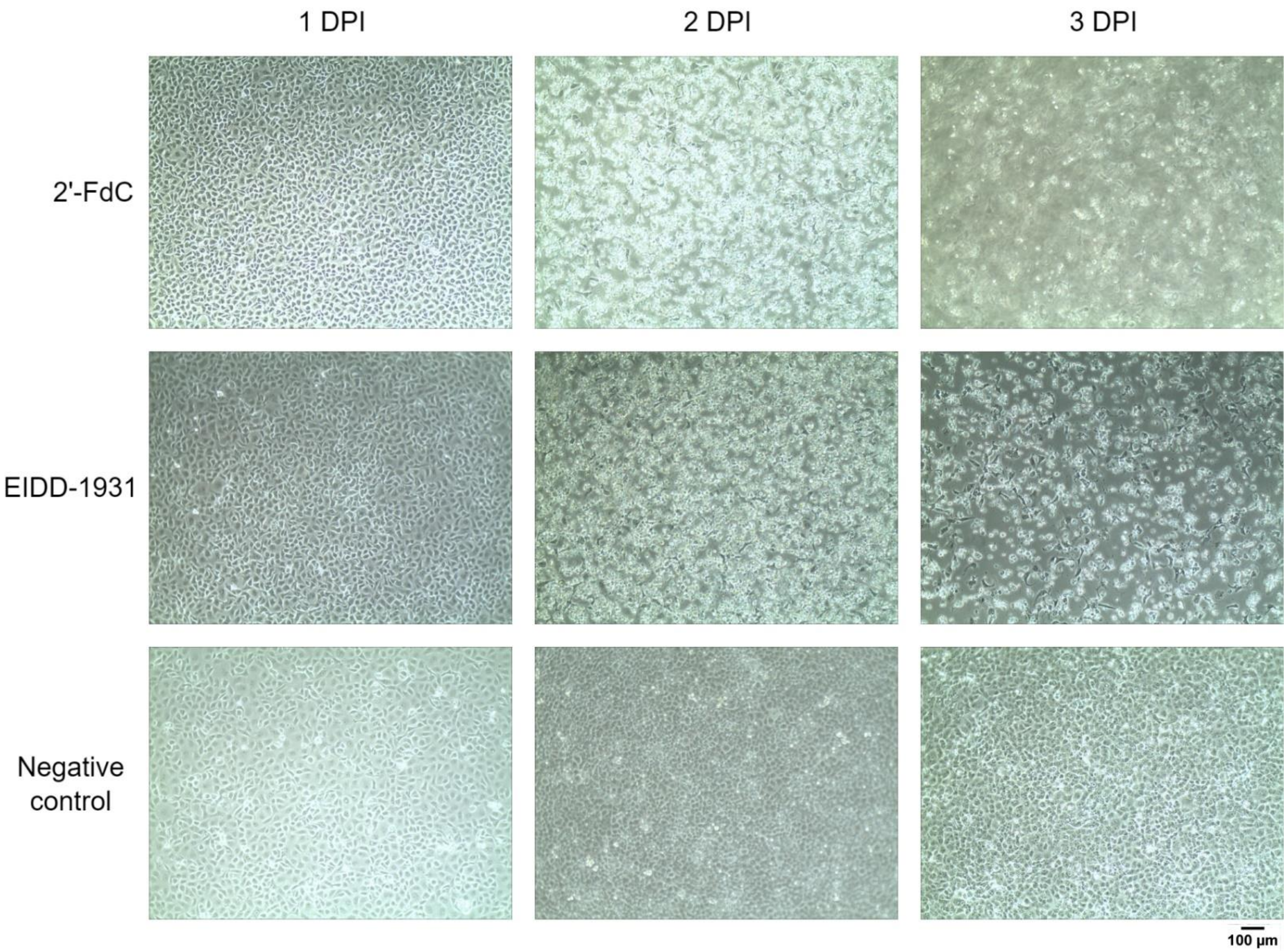

Figure S4. The development of the cytopathic effect on VERO E6 cell culture infected by RVFV, strain ZH-548 (passage 1). Cells were treated with 0.1X fraction of the EC_{50} of respective inhibitors (2´-FdC and EIDD-1931) and incubated until the cytopathic effect covered 90% of the culture. During passage 1, all cultures developed strong cytopathic effect 3 days post-infection (3 DPI). Cells cultivated in the growth media without the presence of the RVFV or inhibitors were used as negative control.

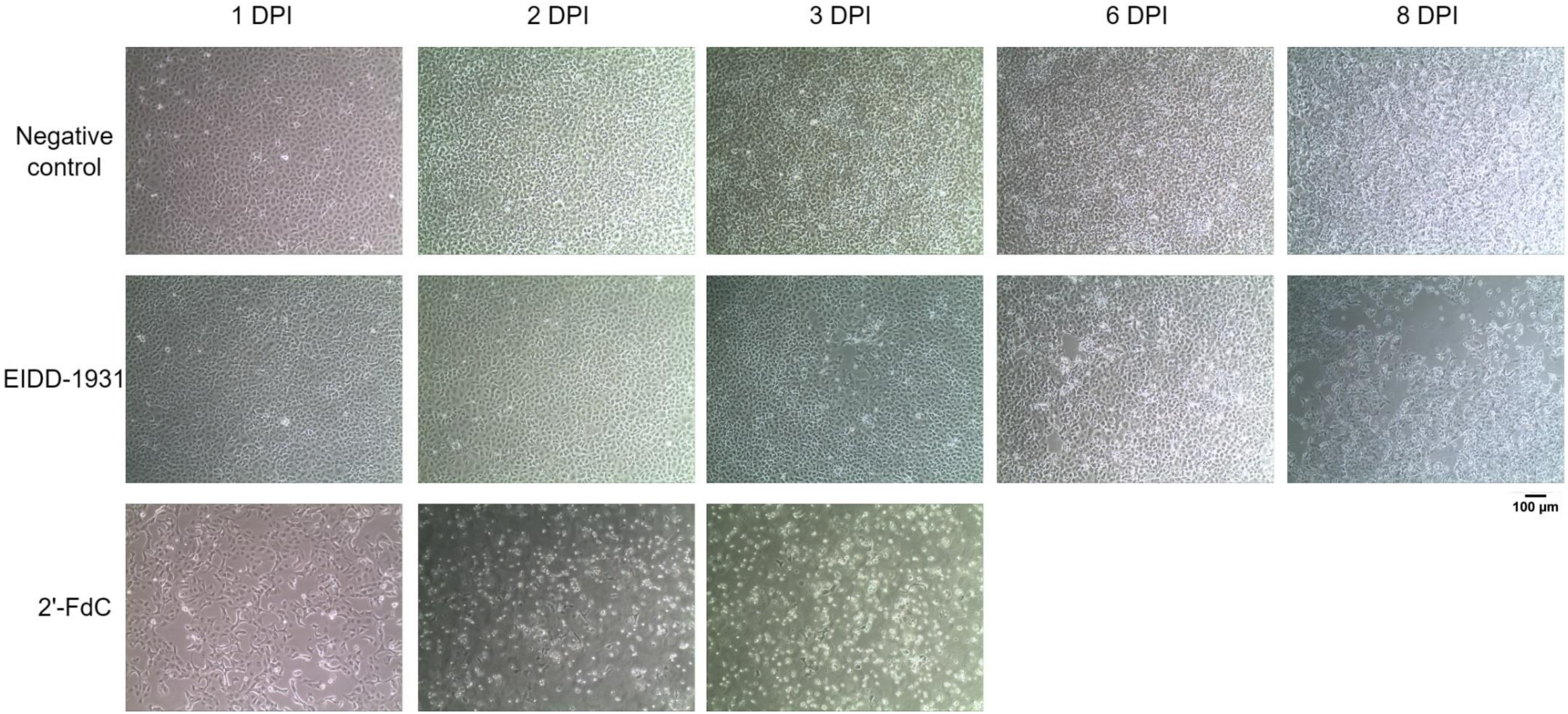

Figure S5. The development of the cytopathic effect on VERO E6 cell culture infected by RVFV, strain ZH-548 (passage 9). Cells were treated with 10X fraction of the EC_{50} of respective inhibitors (EIDD-1931 and 2´-FdC) and incubated until the cytopathic effect covered 90% of the culture. During the last passage, the 2’-FdC-resistant mutant was able to develop strong cytopathic effect 3 days post-infection (3 DPI), similarly to the initial infection in passage 1 (Figure S1). However, EIDD-1931-resistant variant displayed greatly increased time period of the cytopathic effect development, which took up to 8 days post-infection (8 DPI). Cells cultivated in the growth media without the presence of the RVFV or inhibitors were used as negative control.

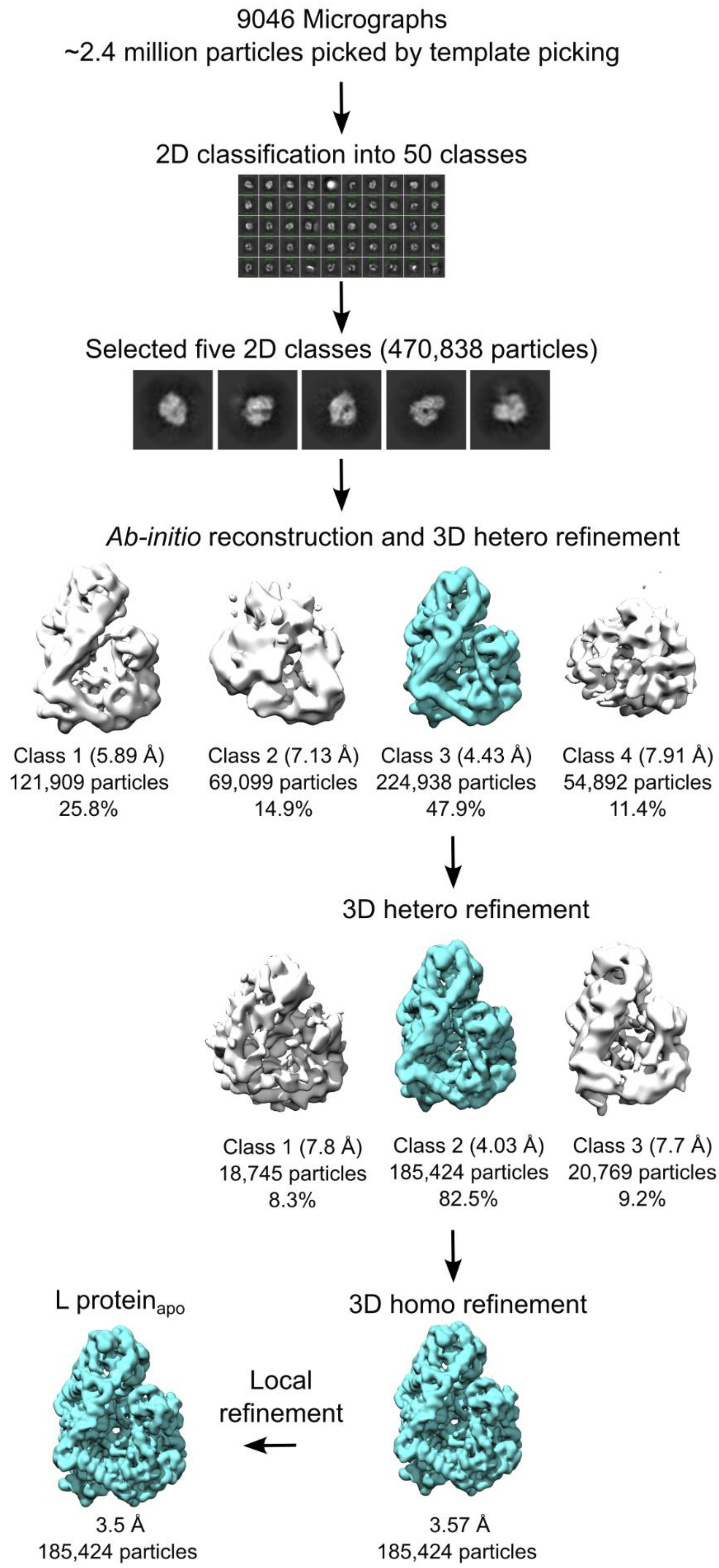

Figure S6. Cryo-EM data classification scheme for the L protein$_{apo}$.

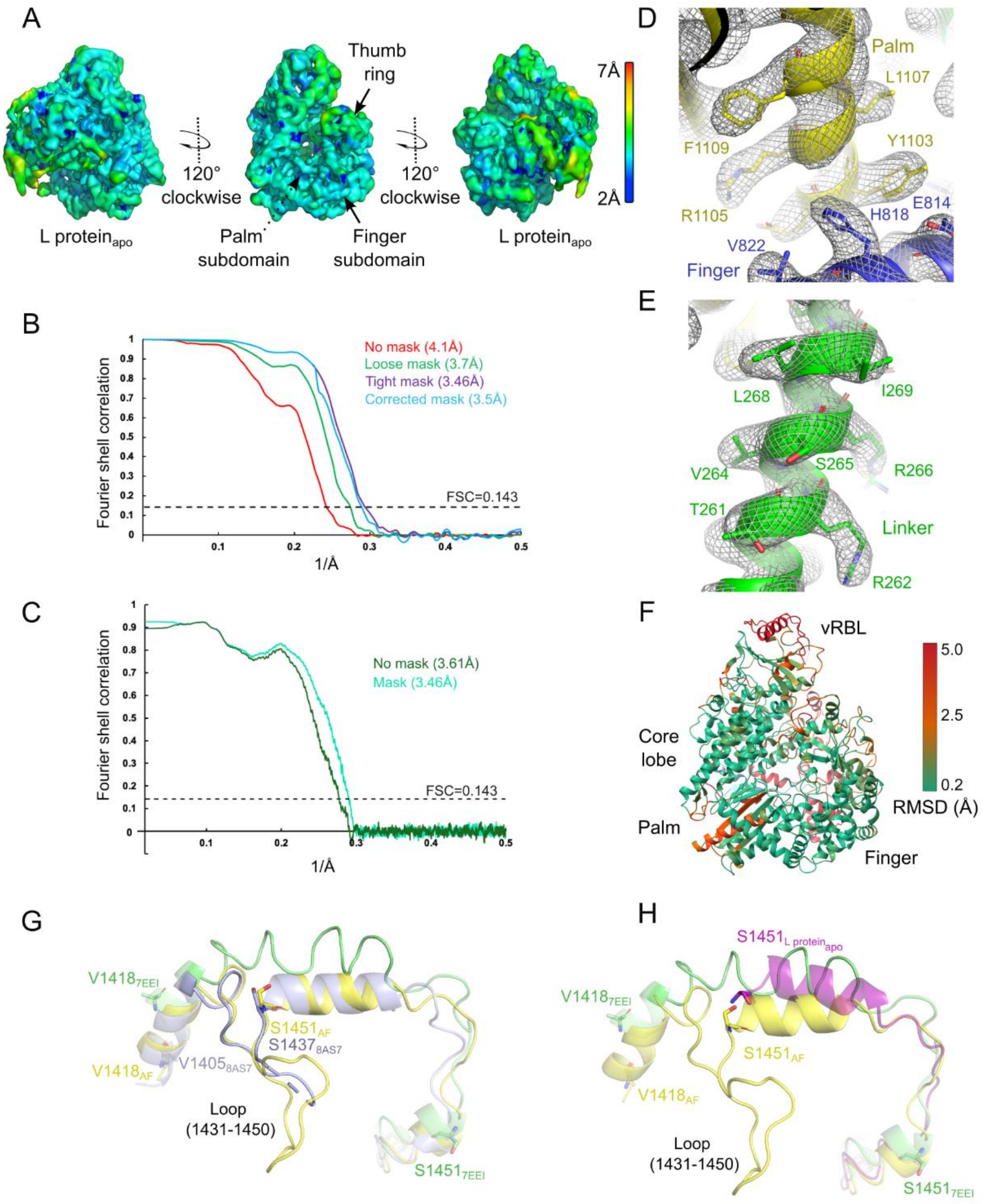

Figure S7. Global and local resolution of the RVFV L protein$_{apo}$ structure, along with its alignment to the previously resolved apo form of the RVFV L protein (PDB 7EEI). (**A**) Surface representation of local resolutions in the L protein$_{apo}$ original unsharpened cryo-EM map. (**B**) Fourier shell correlation (FSC) curves for the final L protein$_{apo}$ cryo-EM map. The FSC plot between two independently refined half maps indicates the overall resolution at ~3.5 Å (FSC cut-off at 0.143). (**C**) FSC between the final experimental cryo-EM density map and the refined atomic model of L protein$_{apo}$. Local map resolution examples for L protein$_{apo}$ are shown highlighting residues from the finger and palm domain (**D**) and the linker region (**E**), colored according to Figure 4B. The L protein$_{apo}$ cryo-EM map (gray mesh) in panels (D) and (E) is shown at σ=6 and was sharpened with a B-factor of -160 Å^2. (**F**) The L protein$_{apo}$ structure, colored according to the root mean square deviation (RMSD) relative to the previously resolved RVFV L protein (PDB 7EEI). (**G**) Superposition of the previous RVFV L protein structure (PDB 7EEI, green), the SFTSV L protein (PDB 8AS7, violet), and the AlphaFold RVFV L protein model (yellow) reveals a loop present in SFTSV and in the AlphaFold model but absent in 7EEI, accounting for the register shift in the earlier RVFV model. (**H**) Superposition of 7EEI (green) with our apo RVFV L protein$_{apo}$ structure (pink) and the AlphaFold model (yellow) further confirms this register shift. In all cases, structures were aligned to the RVFV L protein$_{apo}$ using the main chain Cα atoms.

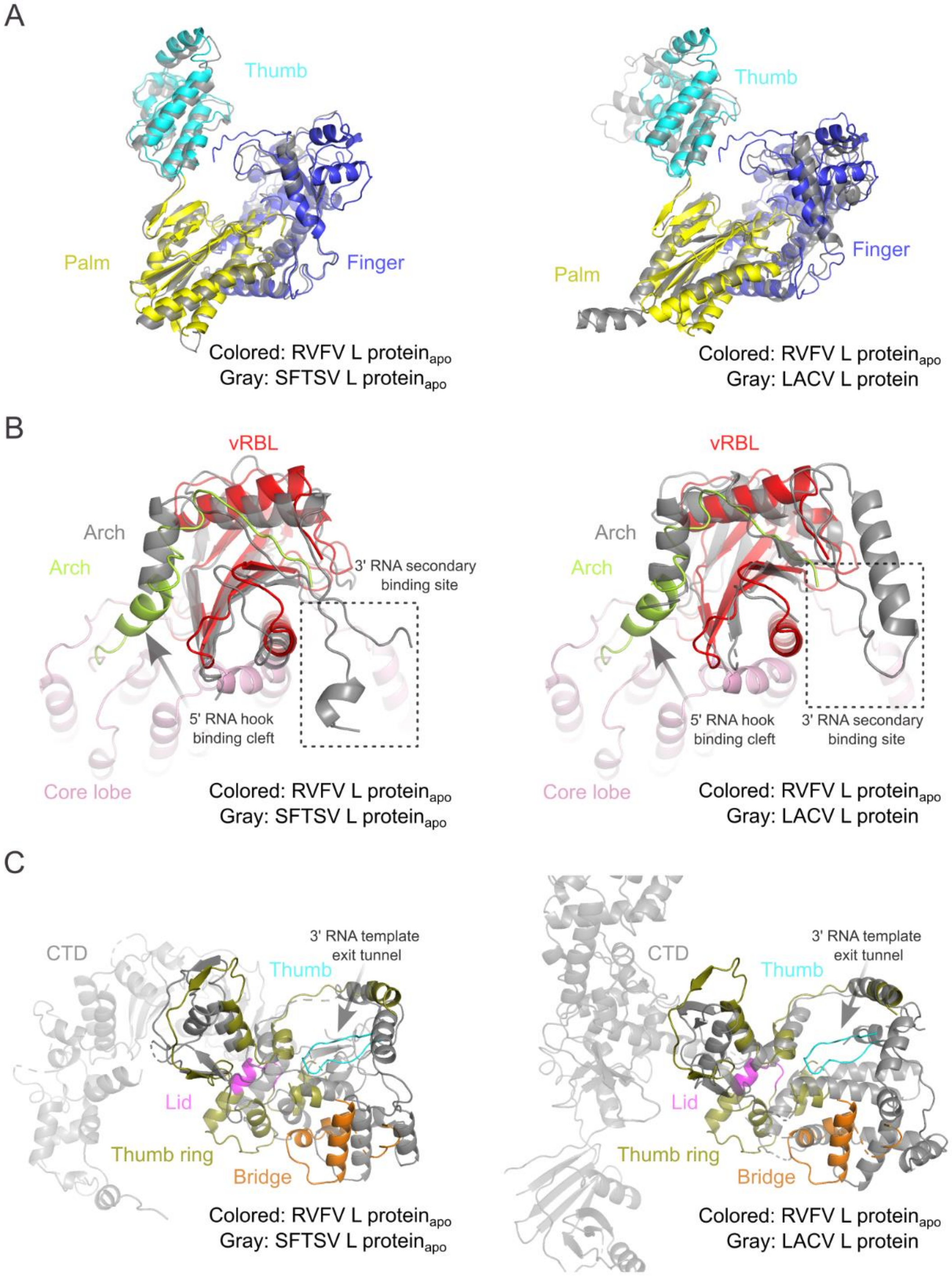

Figure S8. Structural comparison of RVFV L protein$_{apo}$ with SFTSV (strain AH 12) and LACV L proteins. (**A**) Structural conservation of the catalytic core is illustrated, highlighting the preserved architecture among the three L proteins. RVFV L protein$_{apo}$ is color-coded as shown in Figure 4, while the SFTSV (PDB 7ALP) and LACV (PDB 6Z6G) L proteins are displayed in gray. (**B**) Structural alignment highlights the vRBL region, where the 5′ RNA hook-binding cleft (arch motif; indicated by an arrow) adopts a closed conformation in RVFV L protein$_{apo}$. In contrast, the SFTSV (PDB 7ALP) and LACV (PDB 6Z6G) L proteins display a pre-opened cleft configuration. The 3′ RNA secondary binding site is marked with a dashed outline. (**C**) Structural alignment reveals positional shifts in the bridge, lid, and thumb ring subdomains due to the absence of the C-terminal domain (C-term) in RVFV L protein$_{apo}$ when compared to the SFTSV (PDB 7ALP) and LACV (PDB 6Z6G) L proteins. The template exit tunnel is indicated by an arrow. The SFTSV (PDB 7ALP) and LACV (PDB 6Z6G) L proteins were aligned to RVFV L protein$_{apo}$ structure using the main chain Cα atoms.

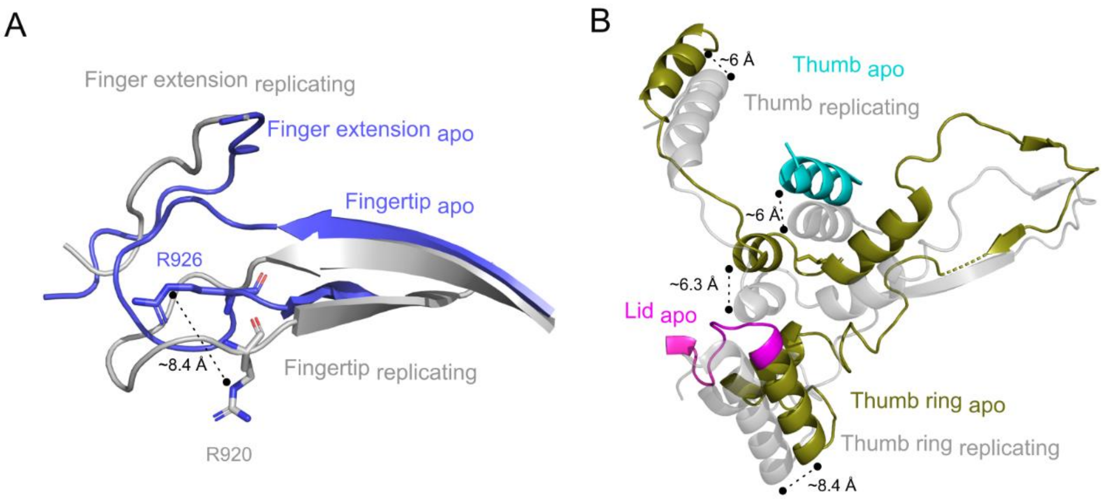

Figure S9. Structural changes between the L protein$_{apo}$ and the SFTSV (strain AH 12) L protein in replication state. (**A**) Structural alignment of the L protein$_{apo}$ (colored according to Figure 4) with the early-stage replicating SFTSV L protein (gray, PDB 8AS7) showing structural rearrangements in the fingertip and finger extension regions. The R926 (analogous to R920 in SFTSV) is positioned far from the active site, whereas R920 during replication in SFTSV is repositioned near the incoming NTP and active site. (**B**) Structural alignment of the L protein$_{apo}$ structure with the early-stage replicating SFTSV L-protein (gray, PDB 8AS7) highlighting the shift (6-8 Å) in the thumb ring and thumb sub-domain critical for the transition from replication initiation to elongation, enabling efficient replication. The SFTSV (PDB 8AS7) L protein was aligned to RVFV L protein$_{apo}$ structure using the main chain Cα atoms.

A

```
              43                    144            164                    749
RVFV   DVSKMVDRITIDFN---GAFQAA-MTKLAKY---VVLYVVSAYRHG---SGRPLLDMQPLI
       DV       +T+D  ---G  +AA  TK  KY---V   V+     G---SGR   +++ ++
SFTSV  DVTVDATGVTVDIG---GGLEAAYRTKIEKY---VFFGVIVVSSGG---SGRST-NLENMV

                           790                                   1667
RVFV   SSLSGMYKK---HLRPQSDAFLG---EKSTANTDF---LFPNIEEL---IYRVLSSV---LSKVLFRTL
         L  MYKK---   P S  FLG---EKST NT +---LFP  +E ---I    S  ---        L
SFTSV  NALGEMYKK---EDKPSSPEFLG---EKSTVNTVY---LFPQAQEY---IIEAFSTL---QVRPFLIFL
```

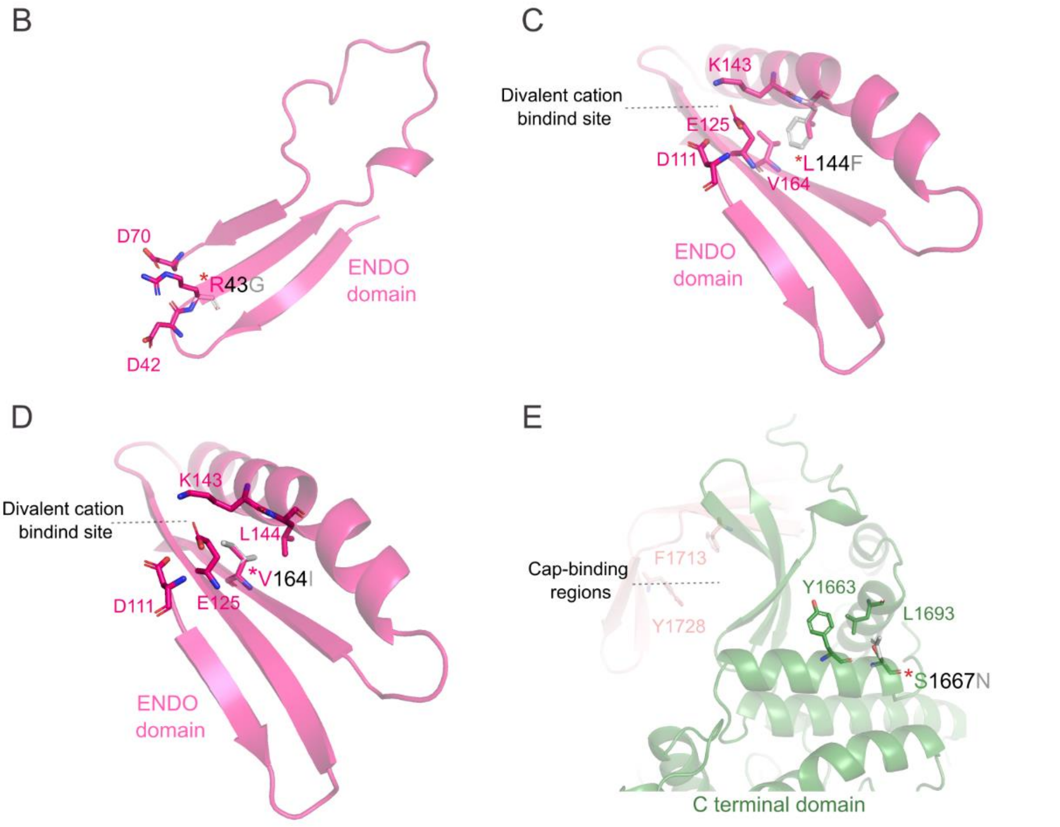

Figure S10. Structural interpretation of the resistance mutations discovered in ENDO and C-terminal domains caused by EIDD-1931. (**A**) Sequence alignment of the RVFV L protein and the SFTSV L protein, highlighting residues that are mutated under selective pressure in RVFV L protein by EIDD-1931. (**B**) Structural AlphaFold model representation of the R43G mutation (sticks) in the ENDO domain (hot pink), highlighting the G43 mutant in gray. (**C**) Structural AlphaFold model representation of the L144F mutation (sticks) in the ENDO domain (hot pink), highlighting the F144 mutant in gray. (**D**) Structural AlphaFold model representation of the V164I mutation (sticks) in the ENDO domain (hot pink), highlighting the I164 mutant in gray. (**E**) Structural AlphaFold model representation of the S1667N mutation (sticks) in the C-terminal domain (forest green), highlighting the N1667 mutant in gray.

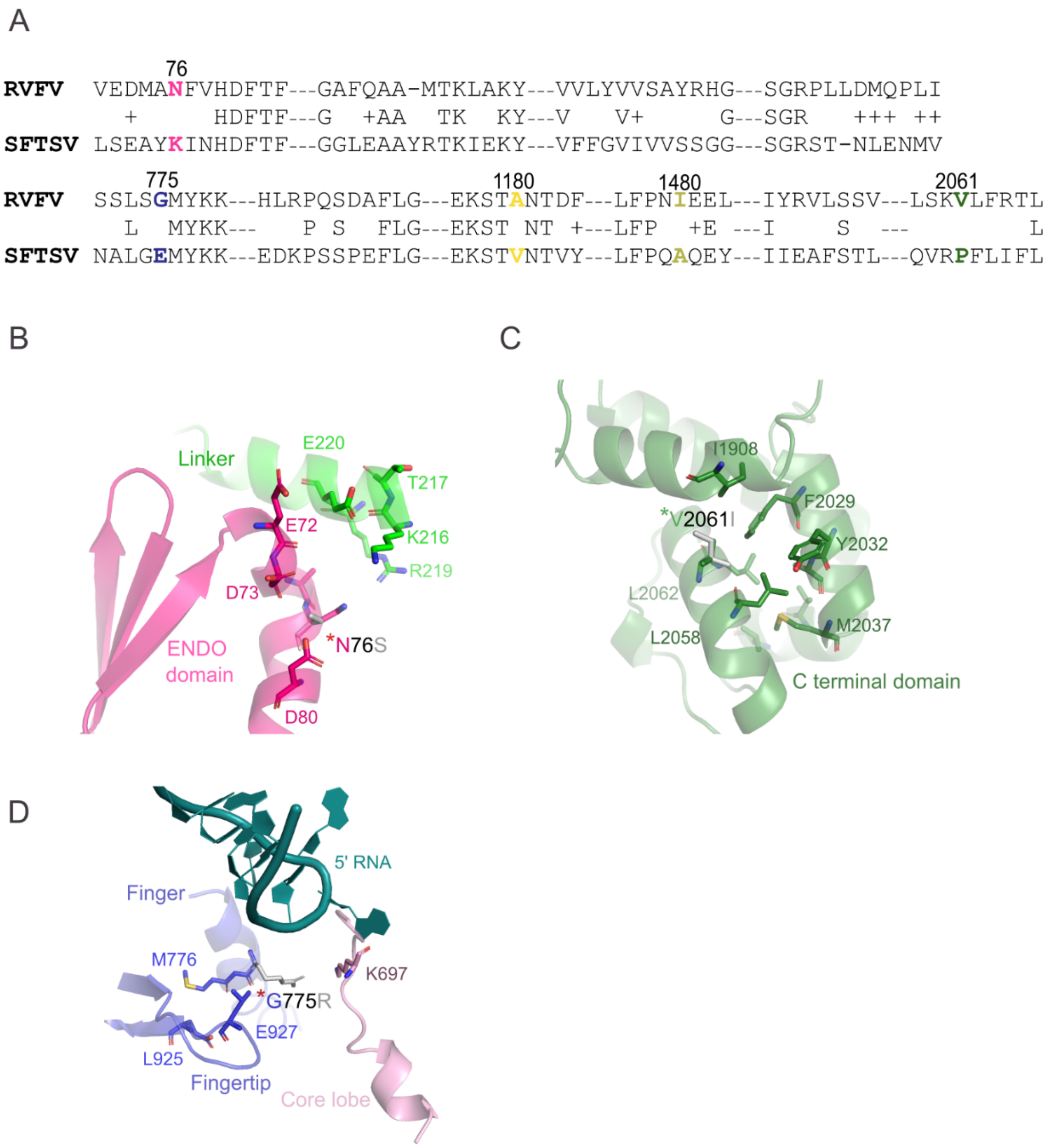

Figure S11. Structural mapping of the resistance mutations identified in the presence of 2`-FdC in the ENDO and C-terminal domains. (**A**) Sequence alignment of the RVFV L protein and the SFTSV L protein, highlighting residues that are mutated under selective pressure by 2`-FdC. (**B**) Structural AlphaFold model showing the ENDO domain (hot pink) with the N76S mutation (sticks), where the S76 mutant is depicted in gray. (**C**) Structural AlphaFold model showing the C-terminal domain with the V2061S mutation (sticks), highlighting the S2061 mutant in gray. (**D**) L protein$_{apo}$ structural model of the G775R mutation (sticks) in the finger sub-domain, with the R775 mutation shown in gray. The 5′ RNA position (marine) is adapted from the replicating SFTSV L protein structure (PDB 8AS7). The fingertip region is modeled from the AlphaFold predicted structure of RVFV L protein, representing the 5′ RNA hook-bound conformation. The structural alignment was based on L protein$_{apo}$ structure using the main chain Cα atoms.

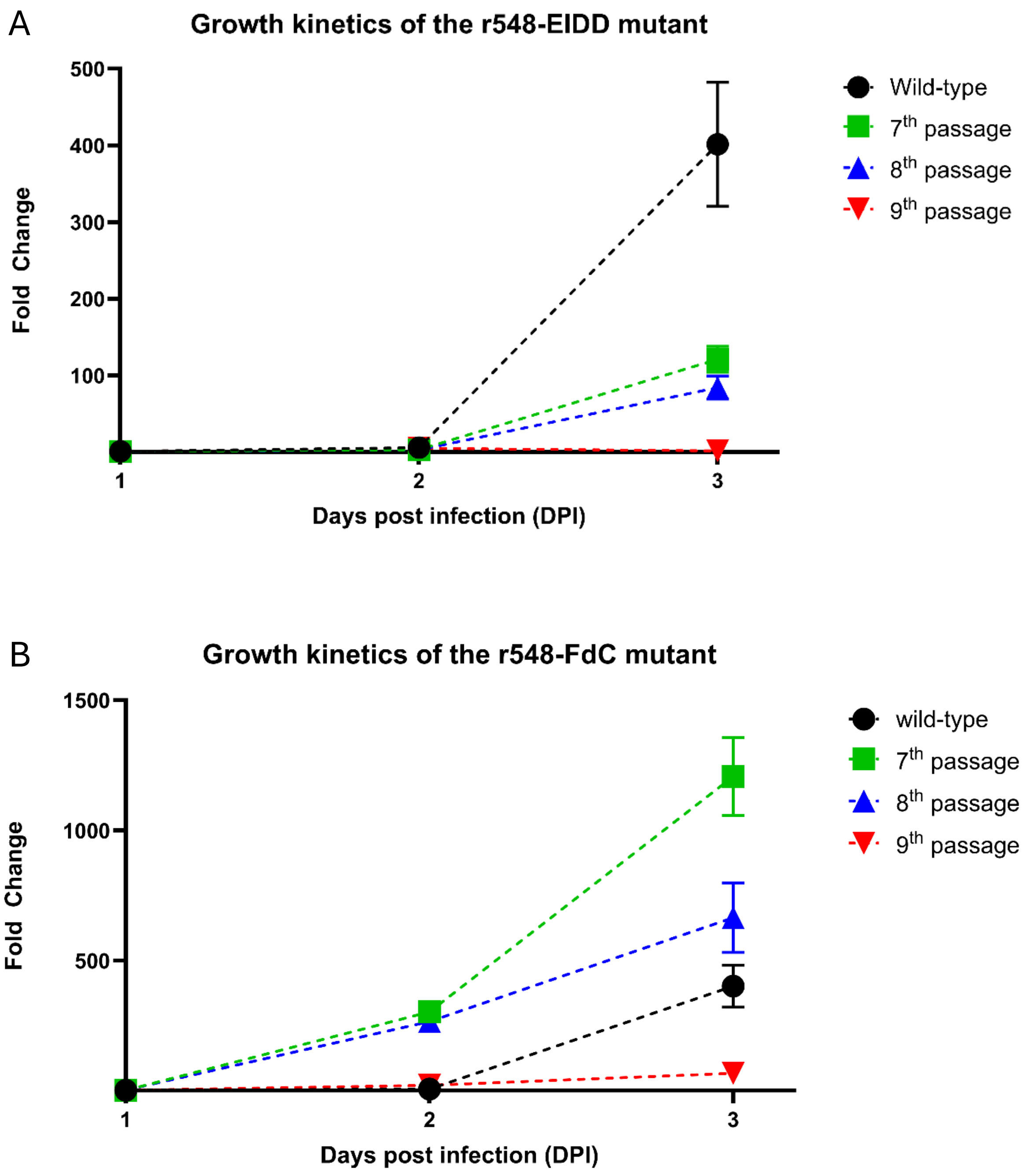

Figure S12. Growth kinetics curves for both wild-type (WT) and resistant variants of 7th, 8th and 9th passages in the presence of EIDD-1931 (**A**) and 2´-FdC (**B**). Final three passages of selection were compared to the growth rate of the wild-type virus. Results were expressed as fold change in viral RNA levels relative to baseline (1 DPI), plotted against days post infection.

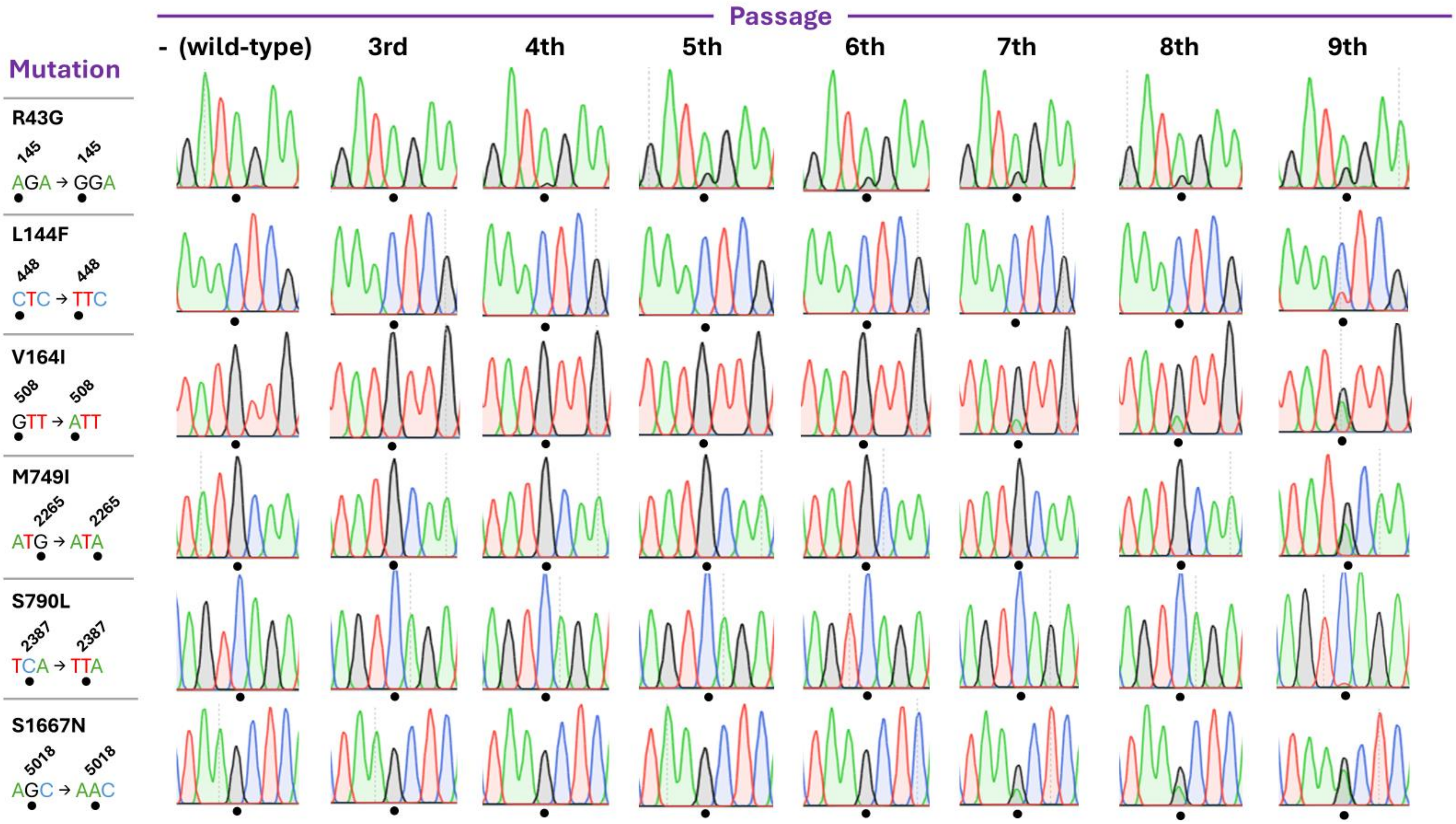

Figure S13. Sanger sequencing analysis reveals progressive emergence of L-segment point mutations in virus passaged with EIDD-1931 compared to wild type. Electropherograms from Sanger sequencing of the viral L-segment are shown for wild-type (unpassaged) virus and for the 3rd through 9th serial passages. Six point non-synonymous mutations are highlighted (R43G, L144F, V164I, M749I, S790L, S1667N), with the corresponding nucleotide substitutions indicated (e.g., AGA→GGA for R43G). Black dots beneath each chromatogram mark the nucleotide positions where mutations are located. The data illustrate the accumulation and persistence of specific mutations across successive passages.

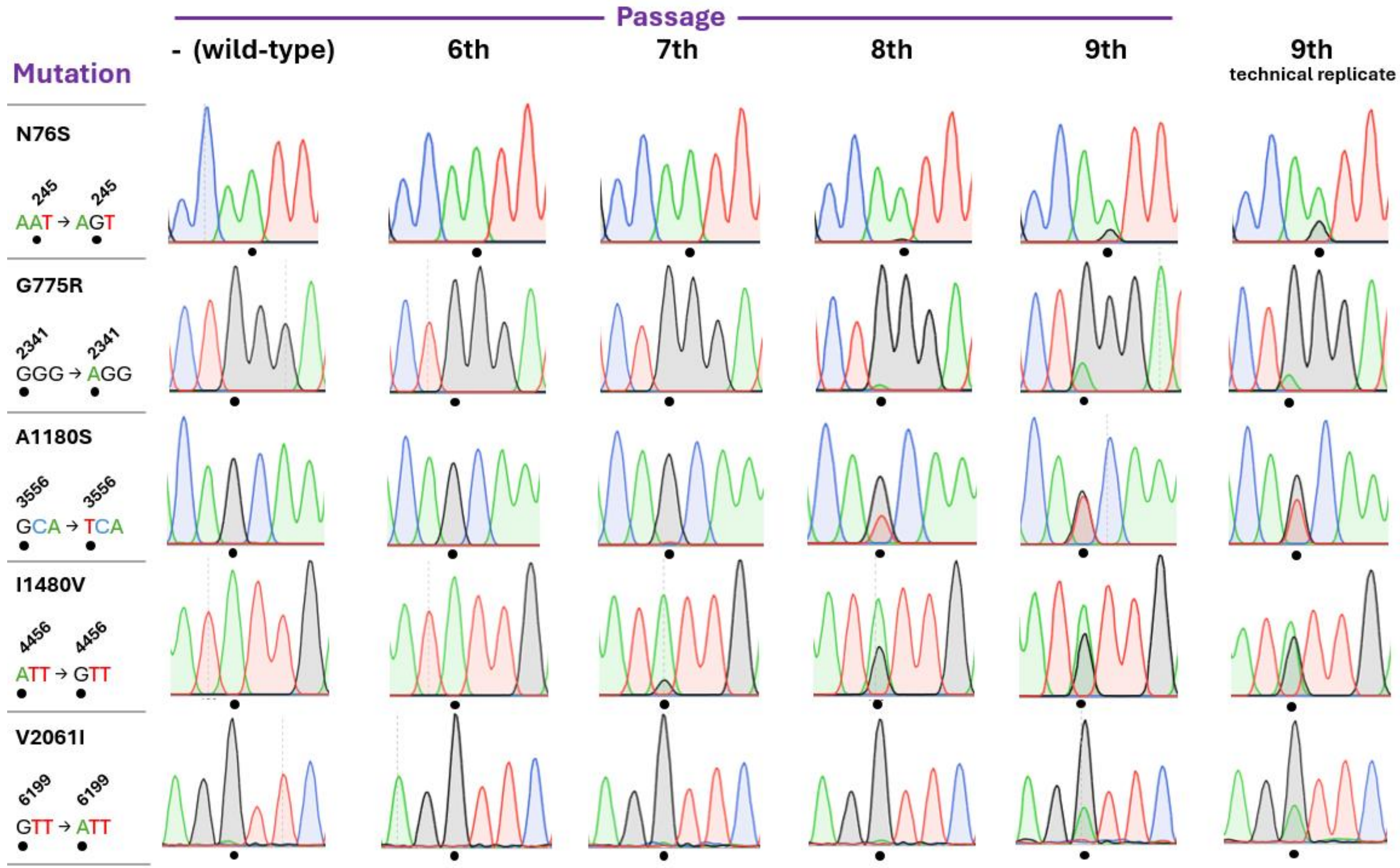

Figure S14. Sanger sequencing analysis reveals progressive emergence of L-segment point mutations in virus passaged with 2´-FdC compared to wild type. Electropherograms from Sanger sequencing of the viral L-segment are shown for wild-type (unpassaged) virus and for the 6th through 9th serial passages. The rightmost column displays a technical replicate of the 9th passage, sequenced using a newly prepared amplicon and alternative primers (Eval1–5). Five point non-synonymous mutations are highlighted (N76S, G775R, A1180S, I1480V, V2061I), with the corresponding nucleotide substitutions indicated (e.g., AAT→AGT for N76S). Black dots beneath each chromatogram mark the nucleotide positions where mutations are located. The data illustrate the accumulation and persistence of specific mutations across successive passages, confirming their reproducibility in technical replication.
